# Poly I:C-induced maternal immune activation causes schizophrenia-like behaviors in the offspring of both sexes by regulating gut microbiota and tryptophan metabolism pathway

**DOI:** 10.3389/fmicb.2025.1667164

**Published:** 2025-09-30

**Authors:** Zhilong Xu, Canrun Hu, Yayan Luo

**Affiliations:** ^1^Institute of Neuropsychiatry, The Affiliated Brain Hospital, Guangzhou Medical University, Guangzhou, Guangdong, China; ^2^Key Laboratory of Neurogenetics and Channelopathies of Guangdong Province and the Ministry of Education of China, Guangzhou Medical University, Guangzhou, China

**Keywords:** Poly I:C, schizophrenia, maternal immune activation, gut microbiota, schizophrenia-like behaviors, tryptophan pathway

## Abstract

Prenatal polyinosinic-polycytidylic acid (Poly I:C) exposure-induced maternal immune activation (MIA) causes schizophrenia-like abnormal behaviors in offspring. Extensive evidence suggests that patients with schizophrenia exhibit gut microbiota dysbiosis and tryptophan (TRP) metabolism dysregulation, which is correlated with psychotic and cognitive symptoms. However, the role of gut microbiota and TRP metabolism in Poly I:C MIA-induced schizophrenia-like behaviors is unclear. In this study, pregnant C57/BL6 mice were injected with Poly I:C (20 mg/kg) or vehicle at gestational day (GD) 9. We found that prenatal Poly I:C exposure at GD 9 led to gut microbiota dysbiosis, thereby activating the TRP-kynurenine (KYN)-quinolinic acid (QA) pathway in the hippocampus, serum, and feces, inhibiting the hippocampal and serum TRP-KYN-kynurenic acid (KYNA) pathway and the hippocampal, serum, and fecal TRP-5-hydroxytryptamine (5-HT) pathway, thus leading to anxiety- and depression-like behaviors and impairments in prepulse inhibition (PPI) and recognition memory in female and/or male offspring during adolescence and/or adulthood. In addition, prenatal Poly I:C exposure caused sex-dependent changes in QA levels and gut microbiota composition in offspring. These results suggest that gut dysbiosis may contribute to prenatal Poly I:C exposure-induced schizophrenia-like behaviors by disturbing the TRP metabolism pathway in adolescent and adult offspring of both sexes. Our study indicates possible strategies for ameliorating prenatal Poly I:C exposure-induced schizophrenia-like behaviors. Our findings provide additional evidence that gut microbiota dysbiosis is an underlying mechanism for Poly I:C MIA-induced schizophrenia-like behaviors and behavioral impairments in schizophrenia. Given the sex-related differences in gut microbiota and QA levels, both sexes should be included in studies that explore the mechanisms of Poly I:C MIA-induced schizophrenia-like behaviors.

## 1 Introduction

Schizophrenia is a chronic and serious psychotic disease characterized by positive, negative, and cognitive symptoms during late adolescence or early adulthood (Abel et al., 2010). This disease exhibits clear sexual dimorphism in age of onset and progression (Abel et al., 2010). Male patients with schizophrenia show a higher rate of diagnosis and earlier age of onset than female patients (Abel et al., 2010). However, the underlying molecular mechanisms of this disorder remain unclear. Epidemiological studies have shown that maternal immune activation (MIA) induced by prenatal infection is a risk factor for the development of schizophrenia in offspring (Sekar et al., 2016). Polyinosinic-polycytidylic acid (Poly I:C), a synthetic analog of double-stranded RNA, binds to Toll-like receptor 3 (TLR3) to activate proinflammatory downstream signaling, leading to increased expression of proinflammatory cytokines and promoting glial cell maturation and differentiation (Bergdolt and Dunaevsky, 2019). Poly I:C exposure during gestation may enhance the risk of schizophrenia in the offspring by inducing MIA (Yildiz Taskiran et al., 2023a,b; Su et al., 2022). Recent studies have found that the offspring of pregnant dams injected with Poly I:C exhibit schizophrenia-like behavioral deficits, such as anxiety- and depression-like behaviors, and deficits in prepulse inhibition (PPI) and recognition memory (Yildiz Taskiran et al., 2023a,b; Su et al., 2022). Therefore, the Poly I:C-induced MIA model has been used to investigate the pathogenesis of schizophrenia (Yildiz Taskiran et al., 2023a,b). However, the molecular mechanism by which Poly I:C-induced MIA causes psychotic and cognitive symptoms in the offspring is not well understood.

The potential role of gut microbiota dysbiosis in schizophrenia has attracted extensive attention in recent years. Previous studies have shown that gut microbiota plays an important role in the development of schizophrenia by leading to abnormalities in nervous system development, metabolic dysfunction, and immune system disorders through the brain-gut axis (Zhu et al., 2020a,b; Guo et al., 2021; Cussotto et al., 2018). Mounting evidence suggests that schizophrenia patients exhibit abnormal composition of gut microbiota that is associated with negative and cognitive symptoms, such as social function, verbal learning, visual learning, working memory, and depression (Zhu et al., 2021; Zhu et al., 2020a,b). In a ketamine mouse model of schizophrenia, mice show an abnormal composition of gut microbiota, which is similar to the microbiome disorder in schizophrenia patients (Xu et al., 2025). In an MK-801 mouse model of schizophrenia, chronic MK-801 exposure for 14 days results in intestinal microbiota dysfunction, anxiety and depression behaviors, and spatial recognition memory impairments, indicating that gut microbiota is associated with these psychotic and cognitive symptoms occurring after chronic MK-801 exposure (Guo et al., 2021). Most importantly, two recent animal studies reported that transplantation of gut microbiota from schizophrenia patients into mice significantly elicits schizophrenia-like behaviors, including psychomotor hyperactivity, learning and memory impairments, and elevated startle responses (Zhu et al., 2020a,b; Zheng et al., 2019). These findings support a crucial role for gut microbiota in the psychotic and cognitive symptoms of schizophrenia.

Tryptophan (TRP) is a biosynthetic precursor of a large number of microbial and host metabolites. TRP is transformed by TRP hydroxylase 1/2 enzyme (TPH1/2) into 5-hydroxytryptophan (5-HTP), which is further metabolized into 5-hydroxytryptamine (5-HT). Another branch of TRP is converted to kynurenine (KYN) by indoleamine 2,3-dioxygenase (IDO). Then, KYN is catabolized by kynurenine aminotransferases (KATs) into kynurenic acid (KYNA), or converted by kynurenine monooxygenase (KMO) to 3-hydroxykynurenine (3-HK), which can be further converted into quinolinic acid (QA) (Agus et al., 2018). TRP metabolic pathways are largely mediated by gut microbiota (Agus et al., 2018). Two previous studies have shown that specific gut microbiota and microbiota metabolites, such as short-chain fatty acids (SCFAs) and secondary bile acids, can stimulate TPH1 expression, which in turn promotes 5-HT synthesis (Yano et al., 2015; Reigstad et al., 2015). As an important neurotransmitter, 5-HT plays a crucial role in the modulation of cognition, immune response, and physiological processes (Yano et al., 2015; Reigstad et al., 2015; Deng et al., 2021). Moreover, gut microbiota could transform TRP into several neuroactive molecules including KYN, KYNA, and QA via stimulating IDO1 (Agus et al., 2018). QA is an N-methyl-D-aspartate (NMDA) receptor agonist that causes excitotoxicity, neurodegeneration, neuroinflammation, and cell damage (Cao et al., 2021). KYNA is an NMDA receptor antagonist that protects against excitotoxic and apoptotic effects and modulates dopaminergic, glutamatergic, and *γ*-aminobutyric acid (GABA)ergic neurotransmission (Cao et al., 2021). An increase in QA and a decrease in KYNA would cause excessive NMDA receptor activation, leading to increased release of reactive oxygen species, resulting in neuronal damage and increased release of glutamate and dopamine, thus impairing brain function (Cao et al., 2021; Agus et al., 2018). In an MK-801 mouse model of schizophrenia, reduced 5-HT levels in the brain are found in mice exposed to MK-801 for 14 days, which are correlated with changes in gut microbiota (Guo et al., 2021). Clinical studies have shown that elevated abundance of gut microbiota in schizophrenia patients is negatively correlated with serum TRP levels and positively correlated with serum KYNA levels (Zhu et al., 2020a,b). A previous study found that schizophrenia patients show a decrease in 5-HT levels in full blood that is negatively correlated with depressive symptoms (Peitl et al., 2016). KYN and KYNA levels are reduced, while QA concentrations are elevated in the serum and plasma of schizophrenia patients; increased QA levels are positively related to cognitive deficits in attention, executive function, language function, and visual learning (Cathomas et al., 2021; Chen et al., 2024). Taken together, these findings suggest that TRP metabolism is a potential mediator between intestinal microbiota and schizophrenia and that gut microbiota may be implicated in schizophrenia by modulating TRP metabolism (Zhu et al., 2020a,b; Guo et al., 2021).

Despite a number of evidences for the involvement of gut microbiota and TRP metabolism in schizophrenia, few studies have investigated the important role of gut microbiota and TRP metabolism in Poly I:C MIA-induced schizophrenia-like psychotic and cognitive symptoms. Two studies have indicated that prenatal Poly I:C exposure at gestational days (GD) 9 and 15 causes abnormal changes in the composition of intestinal flora that may be involved in schizophrenia-like behaviors such as anxiety-like behaviors, recognition memory deficits, and PPI impairments in adult male offspring (Li et al., 2021; Romero-Miguel et al., 2023). Only one study has demonstrated that prenatal Poly I:C exposure increases 3-HK levels in the placenta and fetal brain, which are associated with impaired recognition memory in adult male offspring (Hasegawa et al., 2024). However, the correlations between gut microbiota and Poly I:C MIA-induced schizophrenia-like abnormal behaviors and TRP metabolism are not well explored. It is unclear whether gut microbiota is involved in prenatal Poly I:C exposure-induced schizophrenia-like behavioral alterations by modulating TRP metabolism. As we know, there have been no attempts to study the potential relationship between the gut microbiome and TRP metabolism in Poly I:C MIA-induced schizophrenia-like behaviors. In the present study, the molecular mechanisms underlying schizophrenia-like abnormal behaviors induced by prenatal Poly I:C exposure in offspring were investigated. The aim of this study was to determine whether prenatal Poly I:C exposure at GD 9 caused changes in gut microbiota, elicited TRP metabolism pathway abnormalities, and led to schizophrenia-like behavioral deficits, including anxiety-like behaviors, depression-like behaviors, and impairments in PPI and recognition memory in female and male offspring during adolescence and adulthood. We also determined the correlations between gut microbiota and behavioral parameters and the TRP metabolism pathway.

## 2 Methods and materials

### 2.1 Animals

Male and female C57/BL6 mice aged 6–9 weeks (Guangdong Medical Laboratory Animal Center, Guangzhou, China) were used in this study. Four animals per cage (of the same sex) were housed at room temperature (22–26 °C) under a 12 h light/dark cycle (lights on at 7:00 a.m.) with access to water and food ad libitum. After mating (1 male: 2 females), the females with the presence of vaginal plugs were identified as pregnant; this was considered GD 0. Pregnant mice were randomly divided into control and treatment groups. All experimental procedures involving animals were approved by the Animal Experimentation Ethics Committee of Guangzhou Medical University (Ethics approval number: N2025-29047) and were performed in accordance with the guidelines of the National Institutes of Health on the care and ethical treatment of animals.

### 2.2 Poly I:C administration

Previous studies have demonstrated that descendants whose mothers were injected with Poly I:C at GD 9 subsequently exhibit schizophrenia-like psychotic and cognitive impairments (Hao et al., 2019). In our study, pregnant dams received a single intraperitoneal (i.p.) injection of Poly I:C (20 mg/kg, Sodium salt, Sigma-Aldrich, P1530) or an equal volume of vehicle (Veh) at GD 9 (Juckel et al., 2021). After birth, all pups were kept with their respective dams (*n* = 10 litters for each treatment group) until weaning on postnatal day (PND) 21 and were housed 3–4 per cage according to sex and littermates. Behavioral assessments, sequencing analysis, and TRP metabolism detection were conducted in offspring of both sexes during PND 40 and 60. For each analysis, only one offspring per sex from each litter was employed. One male offspring and one female offspring were randomly selected from each litter for behavioral tests (10 males and 10 females per group), TRP metabolism analysis in serum (8 males and 8 females per group), hippocampus (6 males and 6 females per group), and feces (10 males and 10 females per group), as well as gut microbiota sequencing and analysis (10 males and 10 females per group).

### 2.3 Liquid chromatography tandem mass spectrometry

Blood samples were collected by removing eyeballs. Serum was obtained by centrifuging at 4,000 × g for 15 min at 4 °C, then aliquoted and stored at −80 °C before use. Hippocampal proteins were extracted as described in our previous study (Xu et al., 2025). The expressions of TRP, 5-HT, KYN, KYNA, and QA in serum, as well as the levels of TRP, 5-HT, KYN, and QA in the hippocampus, were detected by Metware Biotechnology Co., Ltd. (Wuhan, China) using LC-MS/MS. LC-MS/MS was conducted with the ExionLC™AD UPLC system and QTRAP^®^ 6,500 + triple quadrupole mass spectrometer equipped with an electrospray ionization source (SCIEX, Inc., Framingham, MA, United States). The mobile phases were water containing 0.1% formic acid (component A) and acetonitrile (component B). The chromatography was performed under multistep gradient conditions at a flow rate of 0.35 mL/min. Multiple Reaction Monitoring was used for quantitative analysis. Data acquisition and analysis were performed using Analyst 1.6.3 and MultiQuant 3.0.3 software.

### 2.4 Enzyme-linked immunosorbent assay

Proteins were extracted from the hippocampus and feces as described in our previous study (Xu et al., 2025). Briefly, the hippocampus and feces were fully homogenized with the appropriate cell lysis buffer. Then, samples were centrifuged at 14,000 × *g* for 15 min at 4 °C, and the supernatants of homogenates were obtained for further measurement. Fecal TRP, 5-HT, KYN, KYNA, and QA levels and hippocampal KYNA concentrations were detected using commercial ELISA kits (Cloud Clone, Wuhan, China; Fine test, Wuhan, China) according to the manufacturers’ instructions. The sensitivities of TRP, 5-HT, KYN, KYNA, and QA were 0.55 μg/mL, 0.5 ng/mL, 4.688 pmol/mL, 0.89 ng/mL, and 0.6 ng/mL, respectively. All measurements were detected in duplicate and expressed as ng/mL. The absorbance was determined using a microtiter plate reader (iMark; Bio-Rad, Hercules, CA, United States) set at 450 nm.

### 2.5 Real-time quantitative PCR

For gene expression experiments, total RNA was extracted from hippocampal tissue using the Mini BEST Universal RNA Extraction Kit (Takara, Shiga, Japan) in accordance with the manufacturer’s protocols. Total RNA was reverse-transcribed into cDNA (37 °C for 15 min and 85 °C for 5 s) using PrimeScript™ RT Master Mix (Takara). RT-qPCR amplification was carried out using the Applied Biosystems ViiA 7 Real-time PCR System (Applied Biosystems, Carlsbad, CA, United States) with the TB Green^®^ Premix Ex Taq™ II (Takara). Thermal cycling conditions were as follows: 50 °C for 2 min; 95 °C for 30 s; and 40 cycles of 95 °C for 5 s, 56 °C for 30 s, and 72 °C for 1 min.

### 2.6 Western blot analysis

Proteins were extracted from the hippocampus using the Tissue Protein Extraction Reagent (T-PER; Thermo Scientific) according to the methods described in our previous study (Luo et al., 2021). Proteins were run on 10% SDS-polyacrylamide gels and transferred to polyvinylidene difluoride membranes (Bio-Rad). The membranes were blocked with 3% bovine serum albumin in Tris-buffered saline for 1 h at room temperature and probed with primary antibodies and horseradish peroxidase (HRP)-conjugated anti-rabbit or anti-mouse secondary antibodies (CST, Boston, United States). Then, the membranes were visualized with SuperSignal West Pico chemiluminescence substrate (Thermo Scientific) and imaged using an imaging system (ChemiDoc XRS+; Bio-Rad).

### 2.7 Fecal microbiota 16S ribosomal RNA sequencing and analysis

Fresh feces were harvested from each mouse at PND 40 and 60, immediately frozen in dry ice individually, and then stored at −80 °C until DNA extraction. Microbial DNA was extracted using a QIAamp DNA Stool Mini Kit (Qiagen, Dusseldorf, Germany) as described by the manufacturer. 16S rRNA sequencing of fecal samples was carried out by Guangdong Magigene Biotechnology Co., Ltd. (Guangzhou, China) as described in our previous study (Xu et al., 2025).

### 2.8 PPI test

The acoustic startle reactivity (ASR) and PPI of the startle reflex were detected using the SR-LAB™ startle response system (San Diego Instruments, San Diego, CA, United States). The mice were allowed to acclimate to the behavioral room for 1 h before the experiment. They were habituated to the startle response chamber with background noise (69 dB) for 5 min. Thereafter, the mice were exposed to 10 presentations of a startling pulse (120 dB, 40 ms duration). The aim of this phase was to promote within-test habituation to startle stimuli. The protocol was consisted 80 trials pseudo-randomly divided into eight trial types as follows: 10 presentations of no pulse, 10 presentations of a startling pulse alone (120 dB, 40 ms duration), 10 presentations of each prepulse alone (76, 79, and 85 dB; 20 ms duration), and 10 presentations of each prepulse with a startling pulse (76 + 120 dB, 79 + 120 dB, and 85 + 120 dB; 100 ms interval). The interval between continuous presentations varied from 10 to 20 s. The percentage of PPI was calculated using the following formula: (ASR amplitude of startling pulse-ASR amplitude of a prepulse with a startling pulse)/(ASR amplitude of a startling pulse) × 100 (Luo et al., 2024). The startle response was assessed by measuring the average ASR amplitude of the startling pulses in every group.

### 2.9 Novel object recognition test

The recognition memory of mice was examined in an open field using a white Plexiglas chamber (50 × 50 × 50 cm) without a lid. Mice were allowed to acclimate to the behavioral room for 1 h before the experiment. They were then placed in the experimental chamber without objects to habituate for 5 min. The novel object recognition test comprised two phases: a 10 min acquisition trial and a 10 min recognition trial, with a 1 h interval between the two trials. During the acquisition trial phase, the mice were allowed to freely explore two identical objects (A, pink polypropylene cubes; length, 3 cm; width, 3 cm; height, 3 cm) placed in opposite corners 15 cm from the walls. During the recognition trial phase, the mice were allowed to freely interact with a familiar object (A) and a novel object (B, yellow polypropylene cylinders; diameter, 3 cm; height, 6 cm). Before the behavioral test, an independent group of mice showed no significant difference in the exploration time between objects A and B (Xu et al., 2025). Behaviors were recorded using Ethovision XT 11.0 video tracking software (Noldus, Wageningen, Netherlands). Exploratory behavior was defined as touching, licking, and sniffing an object. The percentage of exploratory preference was estimated as follows: (time exploring the novel object/total exploration time) × 100 (Luo et al., 2022).

### 2.10 Open field test

The locomotor activity and anxiety-like behavior were measured in an open field with a white Plexiglas chamber (50 × 50 × 50 cm) without a lid. The 20 × 20 cm central section inside the arena is designated as the center of the arena, while the others were designated as periphery. Mice were allowed to acclimate to the behavioral room for 1 h before the experiment. They were then gently placed in the center of the apparatus and allowed to move freely for 5 min. Behaviors were recorded by an EthoVision XT 11.0 video tracking system (Noldus). Time spent in the central area and the number of entries to the center zone were used to measure anxiety-like behavior (Su et al., 2022). Total distance moved was used to evaluate the locomotion of mice (Su et al., 2022).

### 2.11 Elevated plus maze test

The anxiety-like behavior was assessed in the EPM (Reisinger et al., 2016). The EPM was a white Plexiglas cross-shaped maze, elevated 70 cm above the floor, consisting of two open arms and two closed arms. Mice were allowed to acclimate to the behavioral room for 1 h before the experiment. They were then placed in the central platform of the apparatus facing an open arm and allowed to freely explore the maze for 5 min. Behaviors were analyzed by an EthoVision XT 11.0 video tracking system (Noldus). The percentage of time spent in open arms was calculated as: (time spent in open arms/total time spent in open and closed arms) × 100. The percentage of the number of entries to open arms was calculated as: (number of entries to open arms / total number of entries) × 100.

### 2.12 Forced swimming test

The depression-like behavior was evaluated in the FST (Su et al., 2022). Mice were allowed to acclimate to the behavioral room for 1 h before the experiment. They were then placed individually in Plexiglas cylinders (height: 30 cm, diameter: 20 cm) with 15 cm deep water, maintained at 23–25 °C. The test lasted 6 min, and only the data of from the last 4 min were analyzed using the EthoVision XT 11.0 video tracking system (Noldus). Total immobility time was recorded to assess the depression-like behavior of mice. A mouse was considered immobile when it remained in an upright floating position and made only the movements needed to keep its head above the water.

### 2.13 Statistical analysis

Data are shown as mean ± standard error of the mean (SEM). IBM SPSS Statistics for Windows version 25.0 was used for data analysis. The Shapiro-Wilk test and Levene’s test were used to test normality and equal variance between the group values, respectively. In OFT, NOR, EPM, FST, and PPI test (startle response data), differences in behavioral parameters were assessed by two-way analysis of variance (ANOVA) followed by Bonferroni’s post-hoc test. PPI% data from the PPI test were evaluated using repeated-measures three-way ANOVA followed by Bonferroni’s post-hoc test. LC-MS/MS, ELISA, RT-qPCR, Western blot, and microbiota data were analyzed by two-way ANOVA followed by Bonferroni’s post-hoc test. The Ln transformation was applied to microbiota data prior to performing ANOVA. Pearson’s correlations were applied to analyze the correlations between gut microbiota and behavioral parameters and the TRP metabolism pathway. A *p*-value <0.05 was defined as statistically significant.

## 3 Results

### 3.1 Poly I:C MIA induced schizophrenia-like behavioral alterations in adolescent and adult offspring of both sexes

**Figure 1:**
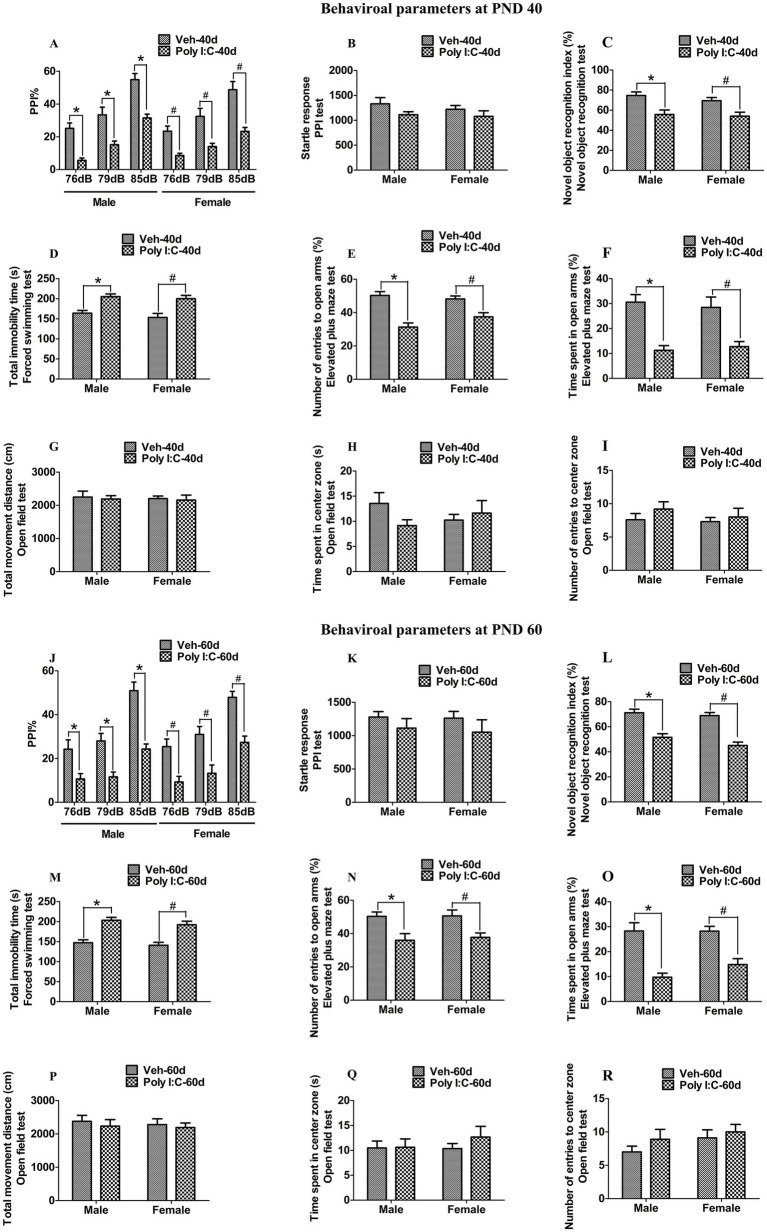
Poly I:C MIA caused schizophrenia-like behavioral changes in the offspring of both sexes during adolescence and adulthood. **(A,J)** A significant reduction in PPI with prepulse intensities of 76, 79, and 85 dB was found in MIA offspring of both sexes at PND 40 and PND 60 (*n* = 10). **(B,K)** There were no significant differences between MIA and Veh offspring of both sexes in terms of startle amplitude in the PPI test at PND 40 and PND 60 (*n* = 10). **(C,L)** A significant decrease in the novel object recognition index was observed in MIA offspring of both sexes at PND 40 and PND 60 (*n* = 10). **(D,M)** Total immobility time in the forced swimming test was significantly elevated in MIA male and female offspring at PND 40 and PND 60 (*n* = 10). **(E,N)** Number of entries to the open arm in the elevated plus maze test was significantly reduced in MIA offspring of both sexes during PND 40 and PND 60 (*n* = 10). **(F,O)** Time spent in the open arms in the elevated plus maze test was significantly decreased in MIA offspring of both sexes during PND 40 and PND 60 (*n* = 10). **(G–I,P–R)** There were no significant differences between MIA and Veh offspring of both sexes in time spent in the center zone, number of entries to the center zone, and total movement distance in the open field test at PND 40 and PND 60 (*n* = 10). Data are expressed as mean ± SEM; * *p* < 0.05 vs. the Veh male, ^#^ *p* < 0.05 vs. the Veh female. Veh, vehicle; MIA, maternal immune activation; PND 40, postnatal day 40; PND 60, postnatal day 60.

We detected the schizophrenia-like behavioral deficits using PPI, NOR, OFT, EPM, and FST in female and male offspring of dams exposed to prenatal Poly I:C at GD 9. As shown in Figures 1A,J, prenatal Poly I:C exposure at GD 9 led to a reduction in PPI with prepulse intensities of 76, 79, and 85 dB in male and female offspring at PND 40 and 60, indicating deficits in sensorimotor gating. As differences in the startle reflex among groups could confound the PPI results, we also measured the startle response. We found no significant differences in startle amplitude in the PPI test between Poly I:C and Veh offspring of both sexes at PND 40 and 60 (Figures 1B,K). Prenatal Poly I:C exposure at GD 9 significantly decreased the novel object recognition index at PND 40 and 60 in offspring of both sexes, indicating impaired recognition memory (Figures 1C,L). The immobility time in the FST was significantly increased in Poly I:C MIA offspring of both sexes during PND 40 and 60, suggesting depression-like behaviors (Figures 1D,M). Prenatal Poly I:C administration at GD 9 reduced the time spent in open arms and the number of entries to open arms in the EPM test, indicating anxiety-like behaviors at PND 40 and 60 in offspring of both sexes (Figures 1E,F,N,O). By contrast, no significant differences in time spent in the center zone, number of entries to the center zone, and total distance moved in the OFT were observed between Poly I:C MIA and Veh offspring of both sexes at PND 40 and 60 (Figures 1G–IP–R).

### 3.2 Poly I:C MIA activated the TRP-KYN-QA metabolism pathway in peripheral serum, hippocampus, and feces of male and female offspring during adolescence and adulthood

To verify whether prenatal Poly I:C exposure altered the TRP metabolism pathway in peripheral serum, hippocampus, and feces of offspring, we used LC-MS/MS and ELISA to detect the expression of TRP, 5-HT, KYN, KYNA, and QA.

**Figure 2:**
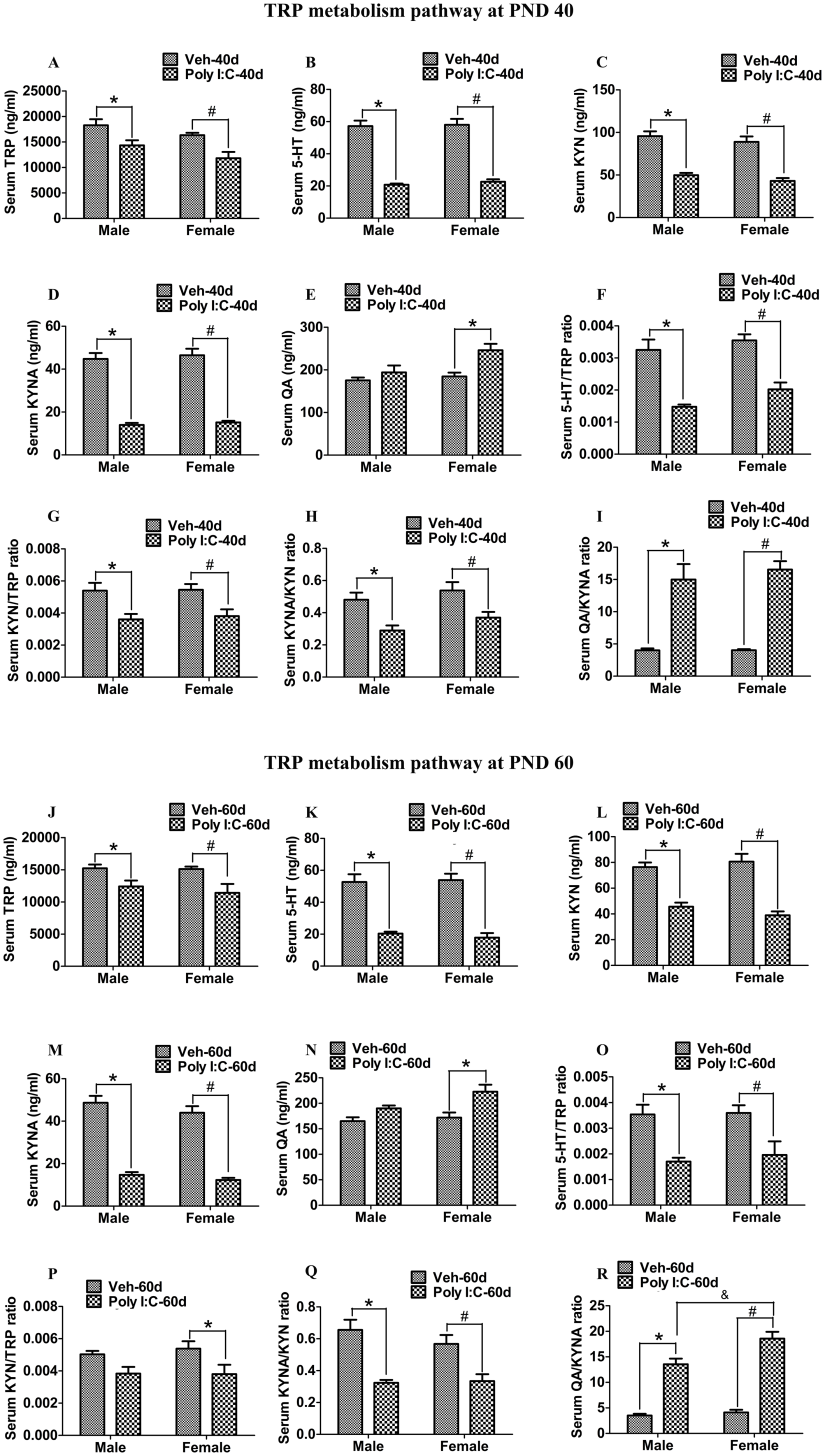
Poly I:C MIA activated the TRP-KYN-QA metabolism pathway in the serum of offspring during adolescence and adulthood. **(A–E,J–N)** Serum TRP, 5-HT, KYN, and KYNA levels were significantly decreased in MIA male and female offspring, while serum QA levels were significantly increased only in MIA female offspring at PND 40 and PND 60. Quantification was determined by LC-MS/MS (*n* = 8). **(F–I)** The ratios of 5-HT/TRP, KYN/TRP, and KYNA/KYN in serum were significantly decreased, while the serum QA/KYNA ratio was significantly increased in MIA male and female offspring at PND 40 (*n* = 8). **(O–Q)** The ratios of 5-HT/TRP and KYNA/KYN in serum were significantly decreased in MIA offspring of both sexes, while serum KYN/TRP ratio was significantly reduced only in MIA female offspring at PND 60 (*n* = 8). **(R)** Serum QA/KYNA ratio was significantly elevated in MIA offspring of both sexes and was higher in females than in males at PND 60 (*n* = 8). Data are expressed as mean ± SEM; * *p* < 0.05 vs. the Veh male, ^#^ *p* < 0.05 vs. the Veh female, ^&^ *p* < 0.05 vs. the Poly I:C male. Veh, vehicle; MIA, maternal immune activation; LC-MS/MS, liquid chromatography tandem mass spectrometry; PND 40, postnatal day 40; PND 60, postnatal day 60.

**Figure 3:**
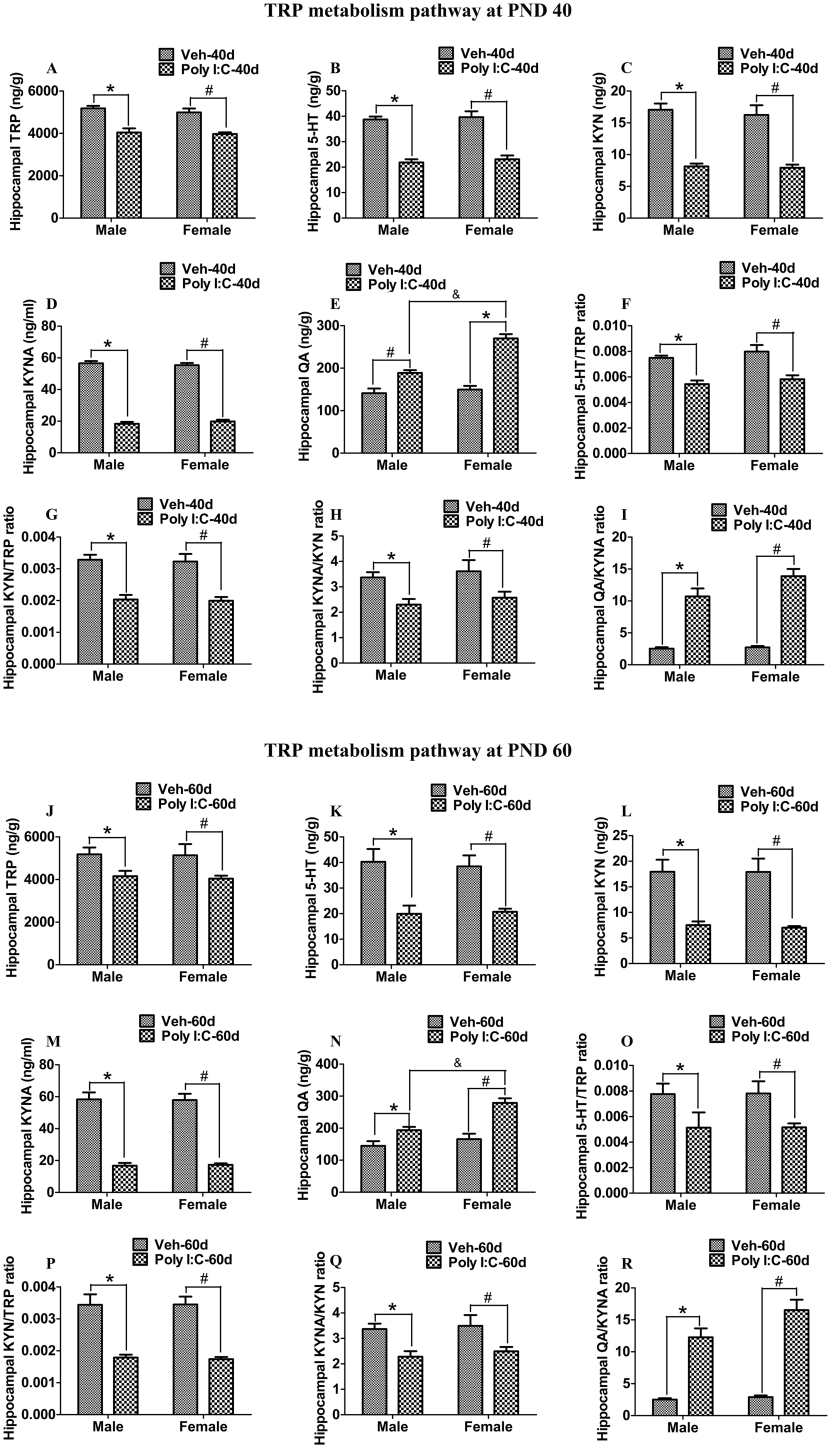
Poly I:C MIA activated the TRP-KYN-QA metabolism pathway in the hippocampus of offspring during adolescence and adulthood. **(A–D,J–M)** Hippocampal TRP, 5-HT, KYN, and KYNA levels were significantly reduced in MIA offspring of both sexes at PND 40 and PND 60. Quantification was determined by LC-MS/MS and ELISA (*n* = 6). **(E,N)** Hippocampal QA levels were significantly enhanced in MIA offspring of both sexes and were higher in females than in males at PND 40 and PND 60. Quantification was determined by LC-MS/MS (*n* = 6). **(F–I,O–R)** The ratios of 5-HT/TRP, KYN/TRP, and KYNA/KYN were significantly decreased, while the QA/KYNA ratio was significantly increased in the hippocampus of MIA male and female offspring at PND 40 and PND 60 (*n* = 6). Data are expressed as mean ± SEM; * *p* < 0.05 vs. the Veh male, ^#^ *p* < 0.05 vs. the Veh female, ^&^ *p* < 0.05 vs. the Poly I:C male. Veh, vehicle; MIA, maternal immune activation; LC-MS/MS, liquid chromatography tandem mass spectrometry; PND 40, postnatal day 40; PND 60, postnatal day 60.

**Figure 4:**
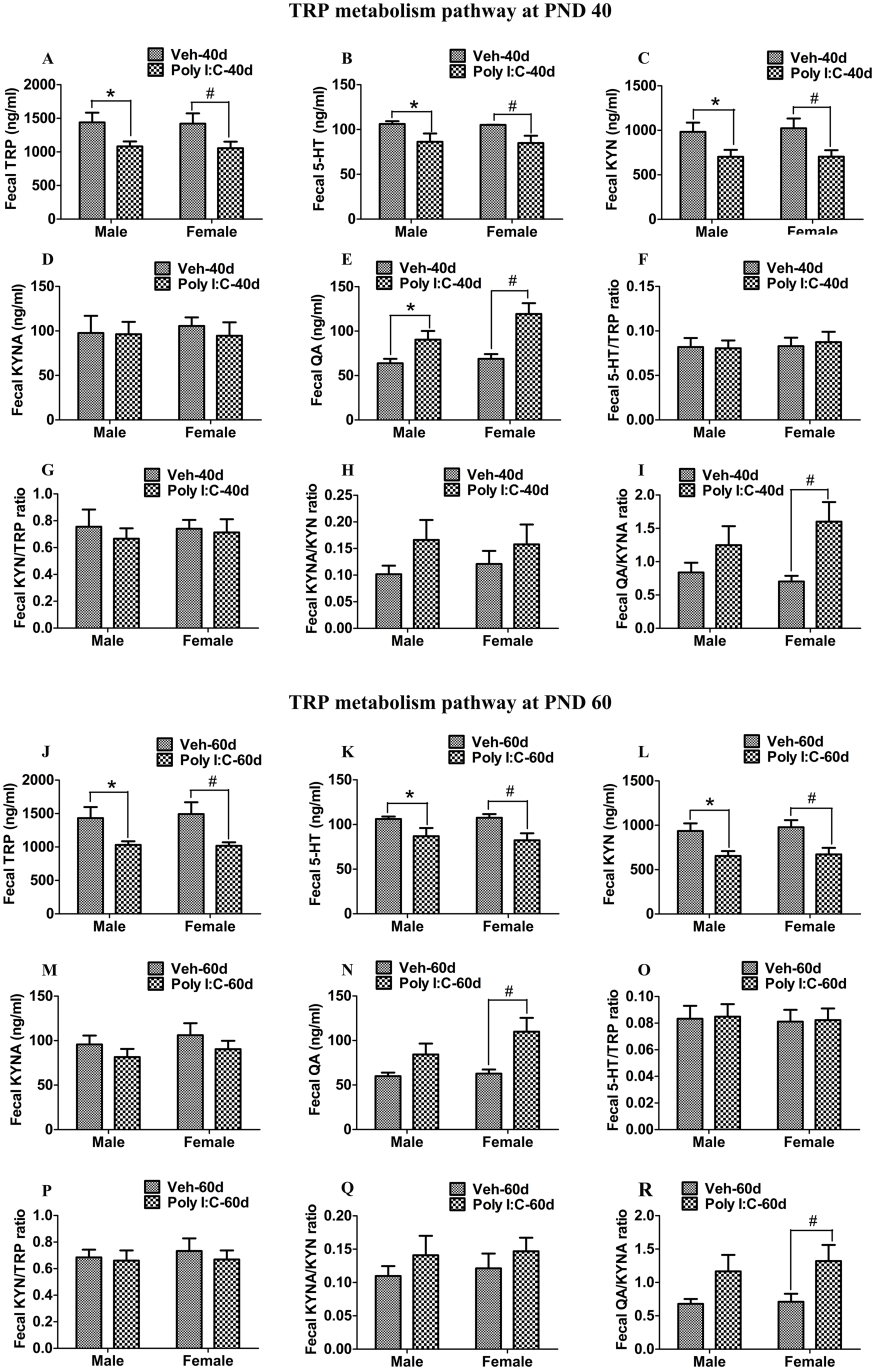
Poly I:C MIA activated the TRP-KYN-QA metabolism pathway in the feces of offspring during adolescence and adulthood. **(A–D,J–M)** Fecal TRP, 5-HT, and KYN levels were significantly reduced, while fecal KYNA levels were unchanged in MIA offspring of both sexes at PND 40 and PND 60. Quantification was determined by ELISA (*n* = 10). **(E,N)** Fecal QA levels were significantly increased in MIA offspring of both sexes at PND 40 and were increased only in MIA female offspring at PND 60. Quantification was determined by ELISA (*n* = 10). **(F–I,O–R)** Fecal 5-HT/TRP, KYN/TRP, and KYNA/KYN ratios were unaltered in MIA offspring of both sexes, while the fecal QA/KYNA ratio was significantly elevated in MIA female offspring at PND 40 and PND 60. (*n* = 10). Data are expressed as mean ± SEM; * *p* < 0.05 vs. the Veh male, ^#^ *p* < 0.05 vs. the Veh female, ^&^ *p* < 0.05 vs. the Poly I:C male. Veh, vehicle; MIA, maternal immune activation; PND 40, postnatal day 40; PND 60, postnatal day 60.

At PND 40, TRP, 5-HT, KYN, and KYNA levels were significantly reduced in both peripheral serum and the hippocampus of male and female offspring of Poly I:C MIA mothers (Figures 2A–D, 3A–D). Serum QA levels were significantly elevated at PND 40 in MIA female offspring (Figure 2E). Hippocampal QA levels were significantly elevated at PND 40 in MIA male and female offspring, and the QA levels were significantly higher in female offspring than in male offspring (Figure 3E). The 5-HT/TRP, KYN/TRP, and KYNA/KYN ratios were significantly decreased, while the QA/KYNA ratio was significantly increased in both peripheral serum and the hippocampus of MIA offspring of both sexes at PND 40 (Figures 2F–I, 3F–I). In addition, the TRP, 5-HT, and KYN levels were significantly reduced, while QA levels were significantly elevated in the feces of MIA offspring of both sexes at PND 40 (Figures 4A–C,E). The KYNA levels were unchanged in the feces of Poly I:C MIA offspring of both sexes at PND 40 (Figure 4D). The fecal QA/KYNA ratio was significantly increased only in MIA female offspring at PND 40 (Figure 4I). There were no significant differences in the ratios of 5-HT/TRP, KYN/TRP, and KYNA/KYN in feces between Poly I:C and Veh offspring of both sexes at PND 40 (Figures 4F–H).

At PND 60, TRP, 5-HT, KYN, and KYNA levels were significantly reduced in both peripheral serum and the hippocampus of male and female offspring of dams administered Poly I:C at GD 9 (Figures 2J–M, 3J–M). Serum QA levels were significantly increased at PND 60 in MIA female offspring (Figure 2N). Hippocampal QA levels were significantly elevated at PND 60 in MIA male and female offspring, and the QA levels were significantly higher in female offspring than in male offspring (Figure 3N). The 5-HT/TRP and KYNA/KYN ratios were significantly reduced at PND 60 in both peripheral serum and the hippocampus of female and male offspring of prenatal Poly I:C-exposed mothers (Figures 2O,Q, 3O,Q). The serum KYN/TRP ratio was significantly decreased at PND 60 in MIA female offspring, while the hippocampal KYN/TRP ratio was significantly decreased at PND 60 in MIA female and male offspring (Figures 2P, 3P). The QA/KYNA ratio was significantly elevated at PND 60 in both peripheral serum and the hippocampus of MIA offspring of both sexes, and the serum QA/KYNA ratio was significantly higher in female offspring than in male offspring (Figures 2R, 3R). In addition, the TRP, 5-HT, and KYN levels were significantly decreased at PND 60 in the feces of MIA offspring of both sexes (Figures 4J–L). Fecal KYNA levels were unchanged in Poly I:C offspring of both sexes at PND 60 (Figure 4M). The QA levels and QA/KYNA ratio were significantly elevated in feces of MIA female offspring at PND 60 (Figures 4N,R). There were no significant differences in the ratios of 5-HT/TRP, KYN/TRP, and KYNA/KYN in the feces of Poly I:C offspring of both sexes at PND 60 (Figures 4O–Q).

### 3.3 Poly I:C MIA significantly altered the expression of TPH2, IDO1, KATII, and KMO in hippocampus of male and female offspring during adolescence and adulthood

**Figure 5:**
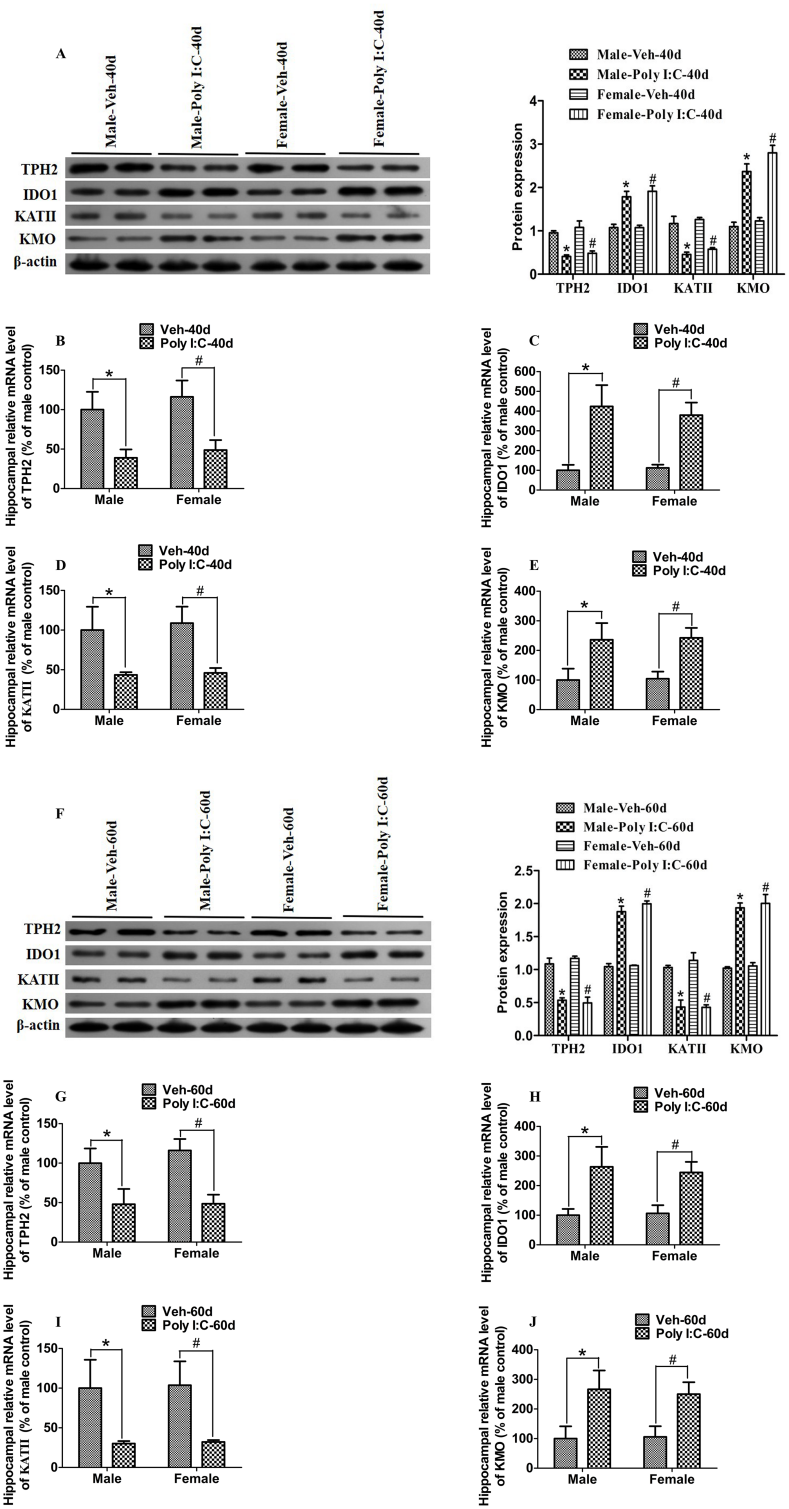
Poly I:C MIA significantly changed the expression of TPH2, IDO1, KATII, and KMO in the hippocampus of male and female offspring during adolescence and adulthood. **(A,F)** The hippocampal protein levels of TPH2 and KATII were significantly reduced, while the hippocampal protein levels of IDO1 and KMO were significantly increased in MIA male and female offspring during PND 40 and PND 60. Left: immunoblot analysis of TPH2, IDO1, KATII, and KMO. Right: quantification of TPH2, IDO1, KATII, and KMO (*n* = 6). **(B–E,G–J)** Hippocampal mRNA levels of TPH2 and KATII were significantly decreased, while hippocampal mRNA levels of IDO1 and KMO were significantly increased in MIA offspring of both sexes at PND 40 and PND 60. Quantification was determined by RT-qPCR (*n* = 6). Data are expressed as mean ± SEM; * *p* < 0.05 vs. the Veh male, ^#^ *p* < 0.05 vs. the Veh female. Veh, vehicle; MIA, maternal immune activation; PND 40, postnatal day 40; PND 60, postnatal day 60.

The TRP metabolism pathway is largely controlled by several rate-limiting enzymes, including TPH2, IDO1, KATII, and KMO. TPH2 and IDO1 are rate-limiting enzymes responsible for the initiation of 5-HT and KYN metabolic pathways, respectively. On the one hand, TRP is metabolized by TPH2 to 5-HTP, which is further converted into 5-HT (Agus et al., 2018). On the other hand, TRP is catabolized by IDO1 into KYN, which is further transformed by KATII to KYNA or by KMO to 3-HK; 3-HK could be further converted into QA (Agus et al., 2018). Therefore, we used RT-qPCR to evaluate changes in the mRNA levels of these key enzymes. At PND 40 and 60, we found that the hippocampal mRNA levels of IDO1 and KMO were significantly elevated, while the hippocampal mRNA levels of TPH2 and KATII were significantly reduced in Poly I:C MIA offspring of both sexes (Figures 5B–EG–J).

Next, we used Western blot analysis to determine the expression of TPH2, IDO1, KATII, and KMO in the hippocampus. As shown in Figures 5A,F, the hippocampal expression levels of TPH2 and KATII were significantly decreased, while the hippocampal expression levels of IDO1 and KMO were significantly increased in Poly I:C MIA offspring of both sexes during PND 40 and 60.

### 3.4 Effect of Poly I:C MIA on the *α*-diversity and *β*-diversity of gut microbiota in offspring of both sexes during adolescence and adulthood

**Figure 6:**
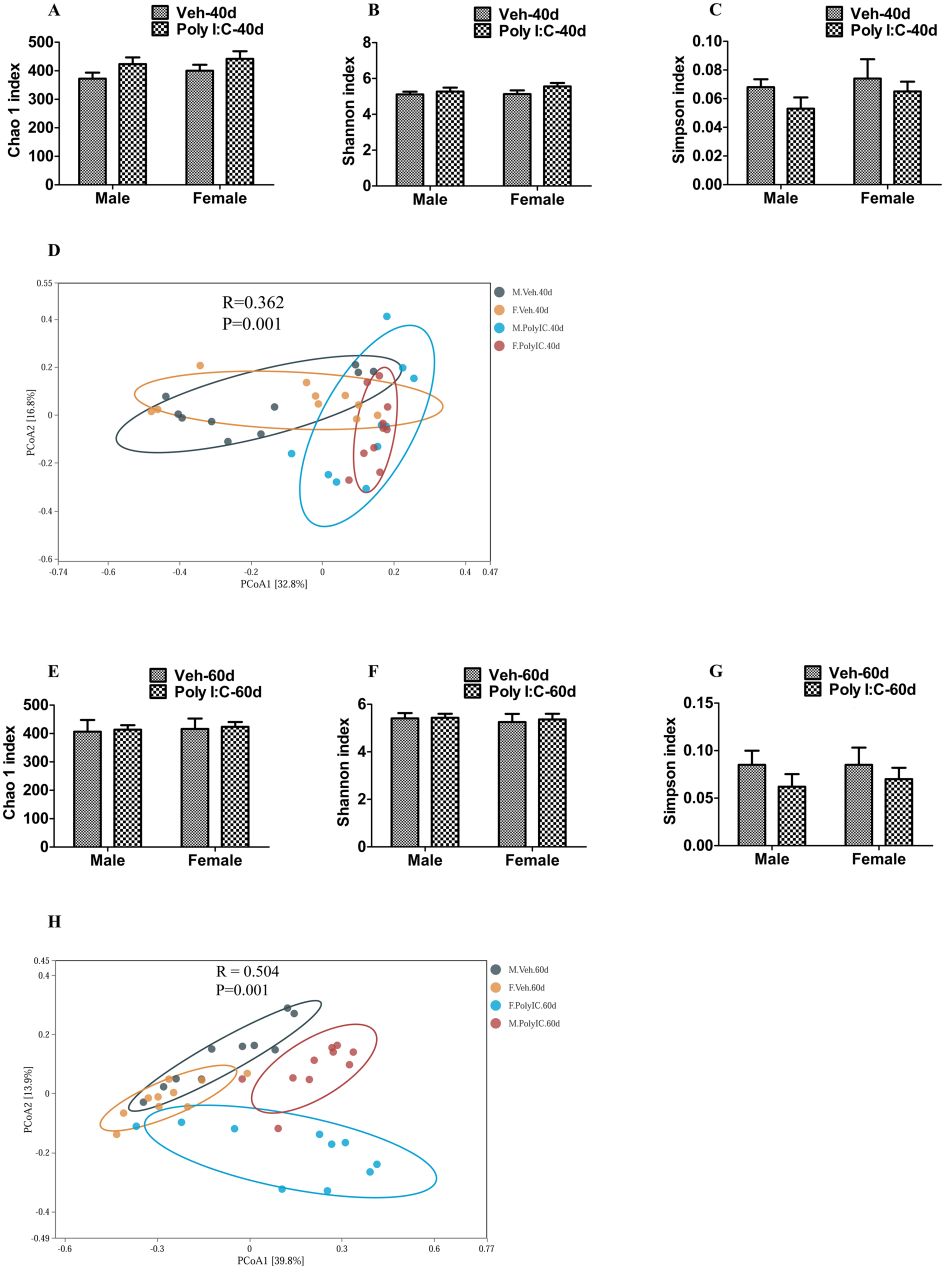
Effect of Poly I:C MIA on *α*- and *β*-diversity of gut microbiota in offspring of two sexes during adolescence and adulthood. **(A–C,E–G)** There were no significant differences in the α-diversity of gut microbiota evaluated by Chao 1, Shannon, and Simpson indices between MIA and Veh offspring of both sexes at PND 40 and PND 60 (*n* = 10). **(D,H)** There were significant differences in β-diversity of gut microbiota estimated by PCoA between the MIA and Veh offspring of both sexes at PND 40 and PND 60 (*n* = 10). Data are expressed as mean ± SEM. Veh, vehicle; MIA, maternal immune activation; PND 40, postnatal day 40; PND 60, postnatal day 60; PCoA, principal coordinate analysis.

We further tested whether prenatal Poly I:C exposure altered the α-diversity and β-diversity of gut microbiota in offspring. At PND 40, we obtained 3, 512, 980 high-quality sequences across all the samples, which were clustered into 813 operational taxonomic units (OTUs) at 97% sequence similarity. A Venn diagram showed that 585 of the 813 OTUs were commonly identified in the four groups, while 16, 23, 29, and 27 were unique to Veh male, Veh female, Poly I:C male, and Poly I:C female at PND 40 in offspring, respectively (Supplementary Figure 1A). At PND 60, a total of 3, 565, 989 valid sequences were obtained from all samples and clustered into 2, 236 OTUs. A Venn diagram showed that 541 of the 2, 236 OTUs were commonly detected among groups, while 340, 155, 28, and 21 were unique to Veh male, Veh female, Poly I:C male, and Poly I:C female at PND 60 in offspring, respectively (Supplementary Figure 1C). The majority of rarefaction curves tended to approach the saturation plateau, suggesting that the sequencing depth was sufficient to cover the whole bacterial diversity at PND 40 and 60 (Supplementary Figures 1B,D). The α-diversity refers to the diversity of bacteria within a community or habitat, quantified by metrics that integrate bacterial richness and evenness (Juckel et al., 2021). Chao 1, Shannon, and Simpson indices are commonly used to estimate the α-diversity of gut microbiota (Xu et al., 2025). In addition, the β-diversity measures the differences in the composition of gut microbiota between groups (Yang P. et al., 2024; Yang Z. et al., 2024). Principal coordinates analysis (PCoA) is a widely utilized indicator for assessing the β-diversity of gut microbiota (Juckel et al., 2021; Yang P. et al., 2024; Yang Z. et al., 2024). In this study, there were no significant differences in the α-diversity of gut microbiota estimated by Chao 1, Shannon, and Simpson indices between MIA and Veh offspring of both sexes at PND 40 and 60 (Figures 6A–CE–G), indicating that prenatal Poly I:C MIA did not significantly change the richness and evenness of gut microbiota. Based on the principal coordinate analysis (PCoA), there were significant differences in the β-diversity of gut microbiota between the Poly I:C and Veh offspring of both sexes at PND 40 and 60 (Figures 6D,H), suggesting that prenatal Poly I:C MIA significantly altered the composition and structure of gut microbiota.

### 3.5 Poly I:C MIA altered the composition of gut microbiota in adolescent and adult offspring of both sexes

**Figure 7:**
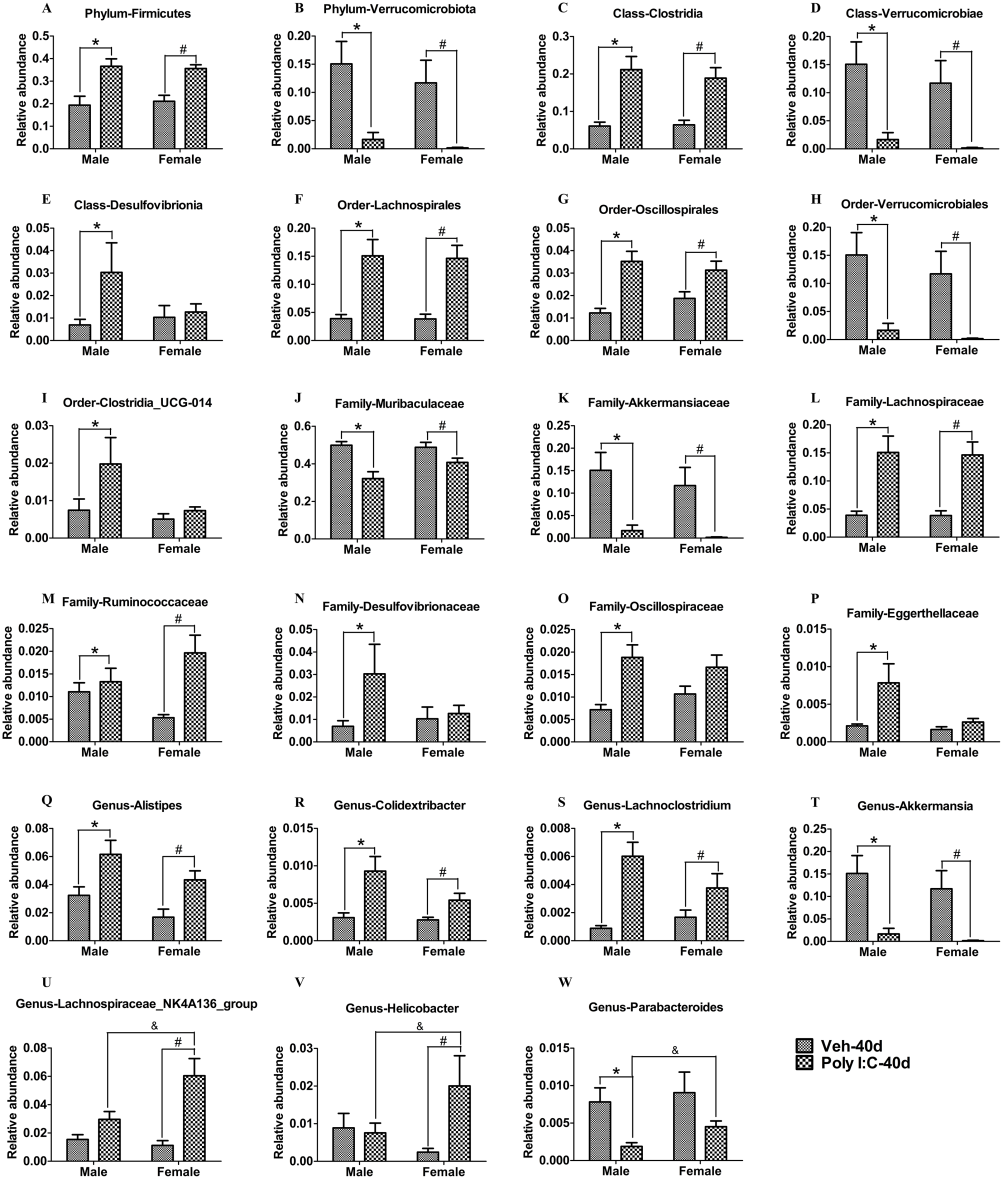
Poly I:C MIA led to changes in the composition of gut microbiota in male and female offspring during adolescence. **(A,B)** At the phylum levels, *Firmicutes* was significantly increased, but *Verrucomicrobiota* was significantly decreased in MIA offspring of both sexes (*n* = 10). **(C,D)** At the class levels, *Clostridia* was significantly elevated, while *Verrucomicrobiae* was significantly reduced in MIA offspring of both sexes (*n* = 10). **(E)** The abundance of *Desulfovibrionia* was significantly increased only in MIA male offspring (*n* = 10). **(F–H)** At the order levels, *Lachnospirales* and *Oscillospirales* were significantly enhanced, while *Verrucomicrobiales* was significantly decreased in MIA male and female offspring (*n* = 10). **(I)** The abundance of *Clostridia_UCG-014* was significantly elevated only in MIA male offspring (*n* = 10). **(J–M)** At the family levels, *Muribaculaceae* and *Akkermansiaceae* were significantly reduced, while *Lachnospiraceae* and *Ruminococcaceae* were significantly enhanced in MIA offspring of both sexes (*n* = 10). **(N–P)** *Desulfovibrionaceae*, *Oscillospiraceae*, and *Eggerthellaceae* were significantly increased only in MIA male offspring (*n* = 10). **(Q–T)** At the genus levels, *Alistipes*, *Colidextribacter*, and *Lachnoclostridium* were significantly increased, but *Akkermansia* was significantly decreased in MIA male and female offspring (*n* = 10). **(U,V)** *Lachnospiraceae_NK4A136_group* and *Helicobacter* were significantly elevated only in MIA female offspring (*n* = 10). **(W)** *Parabacteroides* was significantly decreased only in MIA male offspring (*n* = 10). Data are expressed as mean ± SEM; * *p* < 0.05 vs. the Veh male, ^#^ *p* < 0.05 vs. the Veh female, ^&^ *p* < 0.05 vs. the Poly I:C male. Veh, vehicle; MIA, maternal immune activation.

We further analyzed whether prenatal Poly I:C exposure changed the composition of gut microbiota in offspring. With respect to the PND 40 time point, at the phylum level, *Firmicutes* was significantly increased, while *Verrucomicrobiota* was significantly decreased in Poly I:C offspring of both sexes (Figures 7A,B). At the class level, *Clostridia* was significantly increased, whereas *Verrucomicrobiae* was significantly reduced in Poly I:C offspring of both sexes (Figures 7C,D); *Desulfovibrionia* was significantly elevated only in Poly I:C male offspring (Figure 7E). At the order level, *Lachnospirales* and *Oscillospirales* were significantly enhanced, whereas *Verrucomicrobiales* was significantly decreased in Poly I:C male and female offspring (Figures 7F–H); *Clostridia_UCG-014* was significantly elevated only in Poly I:C male offspring (Figure 7I). At the family level, *Muribaculaceae* and *Akkermansiaceae* were significantly reduced, but *Lachnospiraceae* and *Ruminococcaceae* were significantly elevated in MIA offspring of both sexes (Figures 7J–M); *Desulfovibrionaceae*, *Oscillospiraceae*, and *Eggerthellaceae* were significantly increased only in Poly I:C male offspring (Figures 7N–P). At the genus level, *Alitipes*, *Colidextribacter*, and *Lachnoclostridium* were significantly increased, while *Akkermansia* was significantly decreased in MIA offspring of both sexes (Figures 7Q–T); *Lachnospiraceae_NK4A136_group* and *Helicobacter* were significantly elevated only in Poly I:C female offspring (Figures 7U,V), and *Parabacteroides* was significantly reduced only in Poly I:C male offspring (Figure 7W).

**Figure 8:**
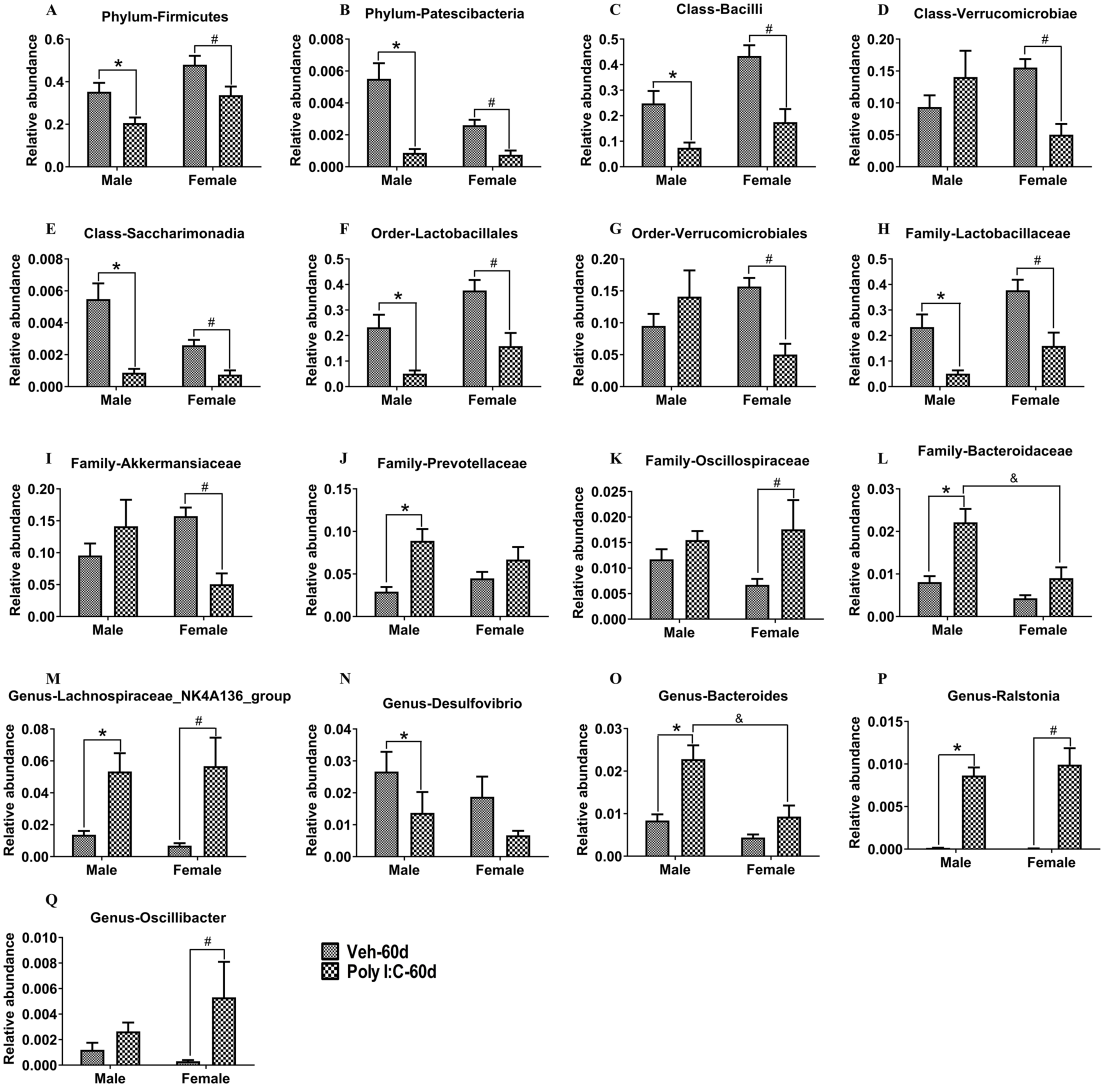
MIA caused alterations in the composition of gut microbiota in male and female offspring at adult period. **(A,B)** At the phylum levels, *Firmicutes* and *Patescibacteria* were significantly decreased in MIA offspring of both sexes (*n* = 10). **(C,E)** At the class levels, *Bacilli* and *Saccharimonadia* were significantly decreased in MIA offspring of both sexes (*n* = 10). **(D)** *Verrucomicrobiae* was significantly reduced only in MIA female offspring (*n* = 10). **(F)** At the order levels, *Lactobacillales* was significantly decreased in MIA offspring of both sexes (*n* = 10). **(G)** *Verrucomicrobiales* was significantly reduced only in MIA female offspring (*n* = 10). **(H)** At the family levels, *Lactobacillaceae* was significantly decreased in MIA offspring of both sexes (*n* = 10). **(I,K)** *Akkermansiaceae* was significantly reduced, while *Oscillospiraceae* was significantly increased only in MIA female offspring (*n* = 10). **(J,L)** *Prevotellaceae* and *Bacteroidaceae* were significantly increased only in MIA male offspring (*n* = 10). **(M,P)** At the genus levels, *Lachnospiraceae_NK4A136_group* and *Ralstonia* were significantly enhanced in MIA offspring of both sexes (*n* = 10). **(N,O)** *Desulfovibrio* was significantly decreased, while *Bacteroides* was significantly increased only in MIA male offspring (*n* = 10). **(Q)** *Oscillibacter* was significantly elevated only in MIA female offspring (*n* = 10). Data are expressed as mean ± SEM; *p < 0.05 vs. the Veh male, ^#^*p* < 0.05 vs. the Veh female, ^&^*p* < 0.05 vs. the Poly I:C male. Veh, vehicle; MIA, maternal immune activation.

With respect to the PND 60 time point, at the phylum level, *Firmicutes* and *Patescibacteria* were significantly decreased in Poly I:C offspring of both sexes (Figures 8A,B). At the class level, *Bacilli* and *Saccharimonadia* were significantly decreased in Poly I:C offspring of both sexes (Figures 8C,E), but *Verrucomicrobiae* was significantly reduced only in Poly I:C female offspring (Figure 8D). At the order level, *Lactobacillales* was significantly decreased in male and female offspring of Poly I:C (Figure 8F), but *Verrucomicrobiales* was significantly reduced only in Poly I:C female offspring (Figure 8G). At the family level, *Lactobacillaceae* was significantly decreased in Poly I:C offspring of both sexes (Figure 8H); *Akkermansiaceae* was significantly reduced, while *Oscillospiraceae* was significantly increased only in Poly I:C female offspring (Figures 8I,K); *Prevotellaceae* was significantly increased only in Poly I:C male offspring (Figure 8J), while *Bacteroidaceae* was significantly increased only in Poly I:C male offspring (Figure 8L). At the genus level, *Lachnospiraceae_NK4A136_group* and *Ralstonia* were significantly enhanced in Poly I:C offspring of both sexes (Figures 8M,P); *Desulfovibrio* was significantly decreased only in Poly I:C male offspring (Figure 8N); *Bacteroides* was significantly increased only in Poly I:C male offspring (Figure 8O); and *Oscillibacter* was significantly elevated only in Poly I:C female offspring (Figure 8Q).

### 3.6 Correlational analysis of gut microbiota with behavioral parameters and TRP metabolism pathway

Finally, we investigated whether changes in gut microbiota were correlated with behavioral parameters and the TRP metabolism pathway in Poly I:C offspring of both sexes during PND 40 and 60. Because prenatal Poly I:C exposure-induced schizophrenia-like abnormal behaviors were evident in both sexes, we focused on those bacterial taxa and TRP metabolites that were similarly altered in Poly I:C male and female offspring.

**Figure 9:**
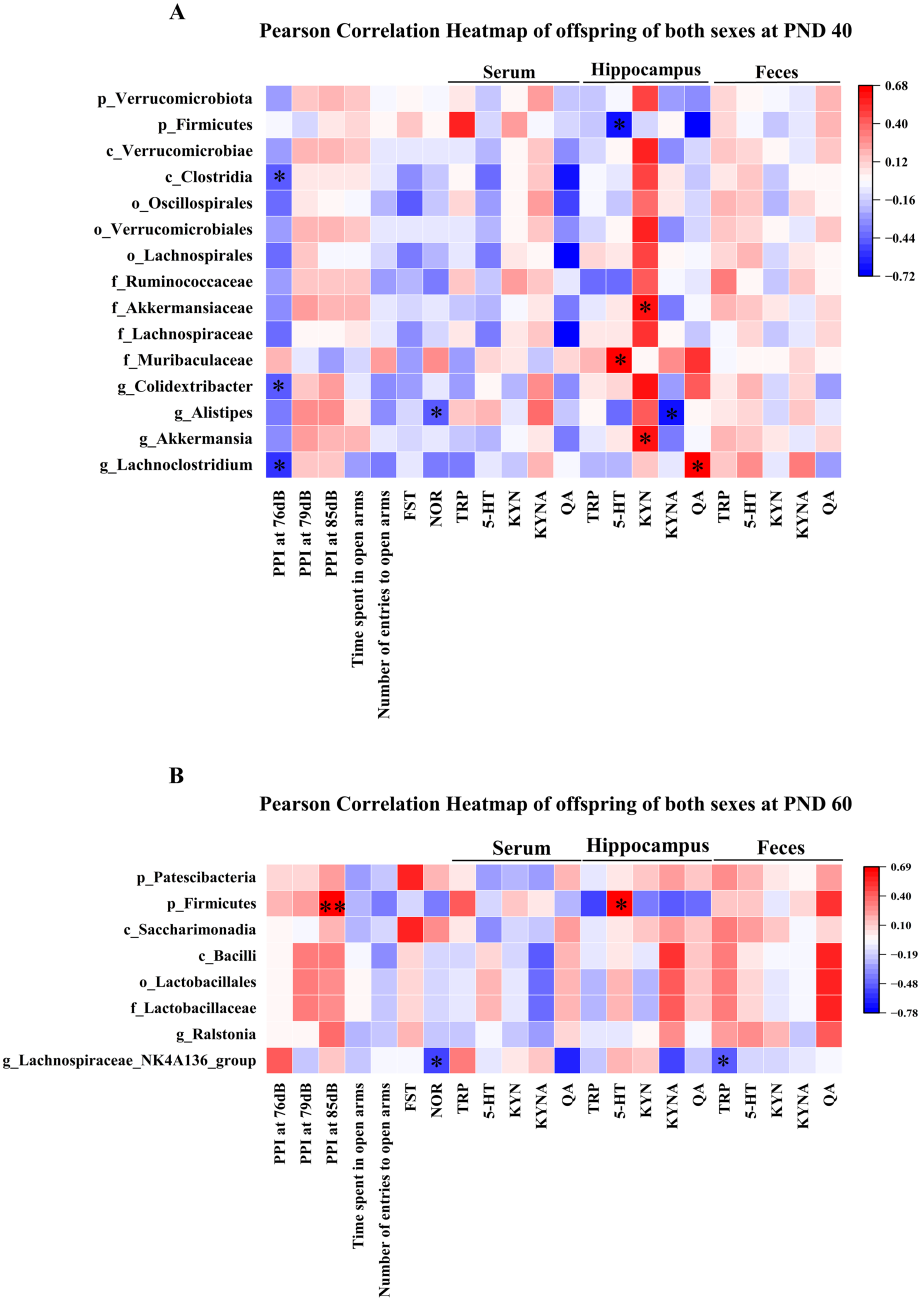
Correlational analysis of gut microbiota with behavioral parameters and the TRP metabolism pathway in MIA offspring of both sexes. **(A)** The heat map showed the correlation of gut microbiota with PPI at 76 dB, novel object recognition index, 5-HT, KYN, KYNA, and QA in MIA offspring of both sexes at PND 40. **(B)** The heat map showed the correlational analysis between gut microbiota and PPI at 85 dB, novel object recognition index, TRP, and 5-HT in MIA male and female offspring during PND 60. Colors ranged from red (positive association) to blue (negative association). * *p* < 0.05, ** *p* < 0.01. p, phylum; c, class; o, order; f, family; g, genus; PND 40, postnatal day 40; PND 60, postnatal day 60.

As shown in Figure 9A, at PND 40, *Clostridia* at the class level and *Colidextribacter* at the genus level were negatively correlated with PPI at 76 dB. *Lachnoclostridium* at the genus level was negatively correlated with PPI at 76 dB and positively correlated with hippocampal QA levels. *Alistipes* at the genus level was negatively associated with the novel object recognition index and hippocampal KYNA levels. *Firmicutes* at the phylum level was negatively associated with hippocampal 5-HT levels. *Akkermansiaceae* at the family level and *Akkermansia* at the genus level were positively correlated with hippocampal KYN levels. *Muribaculaceae,* at the family level showed a positive association with hippocampal 5-HT levels.

As shown in Figure 9B, at PND 60, *Firmicutes* at the phylum level was positively correlated with PPI at 85 dB and hippocampal 5-HT levels. The *Lachnospiraceae_NK4A136_group* at the genus level showed a negative association with the novel object recognition index and fecal TRP levels.

In addition, our results showed that prenatal Poly I:C exposure has different impacts on the composition of gut microbiota in offspring of two sexes, suggesting sex-related differences in gut microbiota composition. Thus, we investigated, separately in Poly I:C MIA male and female offspring, the correlations between gut microbiota alterations and behavioral parameters as well as the TRP metabolism pathway at PND 40 and 60.

**Figure 10:**
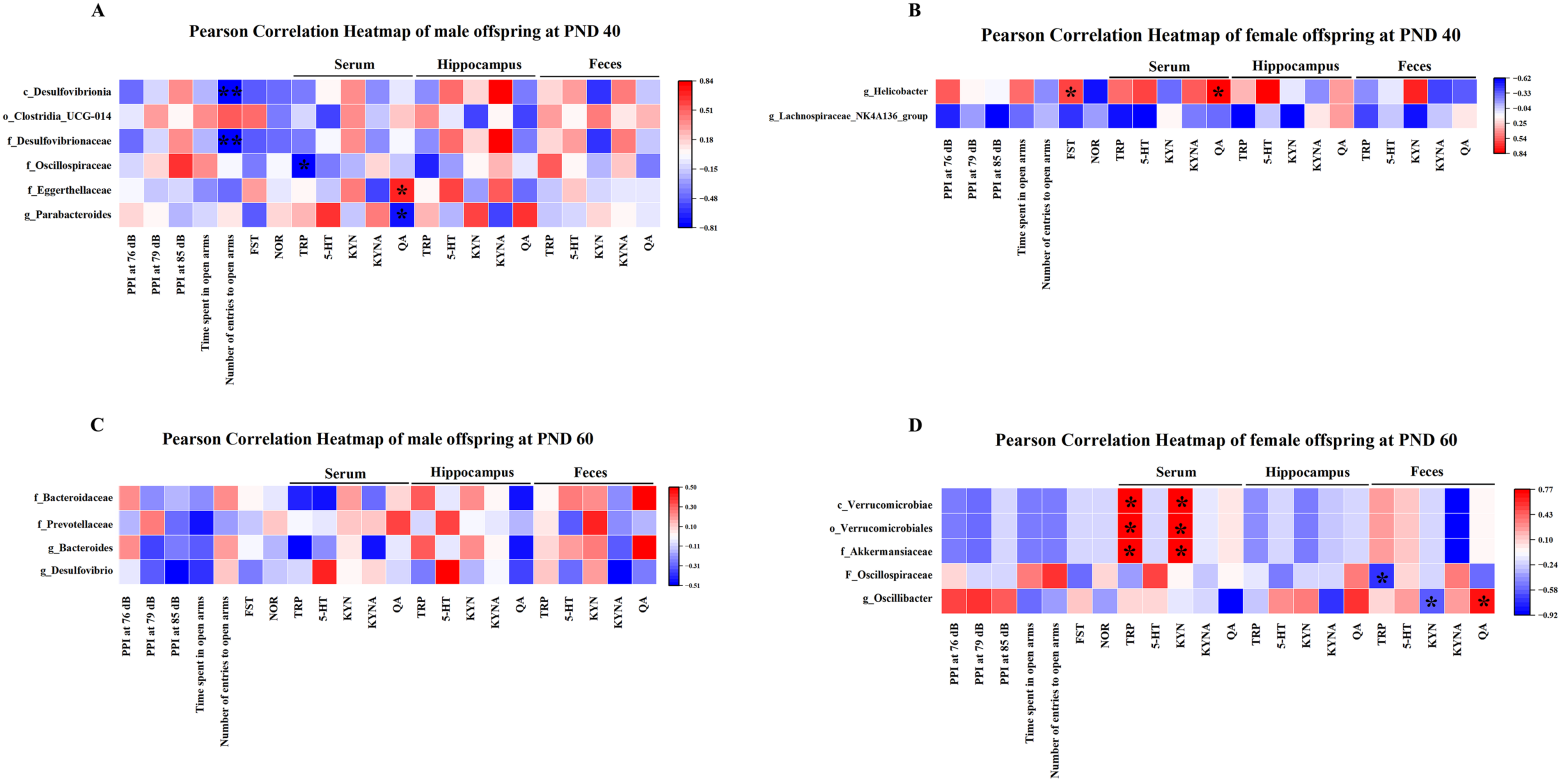
Correlational analysis of gut microbiota with behavioral parameters and the TRP metabolism pathway separately in MIA males and females. **(A)** The heat map revealed the correlation of the gut microbiota with the number of entries to open arms, TRP, and QA in MIA male offspring at PND 40. **(B)** The heat map revealed the association between the gut microbiota and immobility time of FST as well as QA in MIA female offspring at PND 40. **(C)** The heat map showed the associational analysis in MIA male offspring at PND 60. **(D)** The heat map showed the correlation of the gut microbiota with TRP, KYN, and QA in MIA female offspring at PND 60. Colors ranged from red (positive association) to blue (negative association). *p < 0.05, **p < 0.01. c, class; o, order; f, family; g, genus; PND 40, postnatal day 40; PND 60, postnatal day 60.

At PND 40, *Desulfovibrionia* at the class level and *Desulfovibrionaceae* at the family level were negatively correlated with the number of entries to open arms in male offspring (Figure 10A). *Oscillospiraceae* at the family level was negatively correlated with serum TRP levels in male offspring (Figure 10A). *Eggerthellaceae* at the family level was positively associated with serum QA levels, while *Parabacteroides* at the genus level was negatively associated with serum QA levels in male offspring (Figure 10A). In addition, *Helicobacter* at the genus level was positively related to the immobility time of the FST and serum QA levels in female offspring (Figure 10B).

At PND 60, there was no significant correlation between gut microbiota and behavioral parameters and TRP metabolic pathways in male offspring (Figure 10C). In addition, *Verrucomicrobiae* at the class level, *Verrucomicrobiales* at the order level, and *Akkermansiaceae* at the family level were positively associated with serum TRP and KYN levels in female offspring (Figure 10D). *Oscillospiraceae* at the family level showed a negative correlation with fecal TRP levels in female offspring (Figure 10D). Moreover, *Oscillibacter* at the genus level was negatively related to fecal KYN levels and positively related to fecal QA levels in female offspring (Figure 10D).

## 4 Discussion

In the present study, we found that prenatal Poly I:C exposure at GD 9 induced gut microbiota dysbiosis, thereby activating the TRP-KYN-QA pathway in hippocampus, serum, and feces, suppressing the hippocampal and serum TRP-KYN-KYNA pathways and the hippocampal, serum, and fecal TRP-5-HT pathways, and thus resulting in anxiety- and depression-like behaviors and deficits in PPI and recognition memory in female and/or male offspring during adolescence and/or adulthood. In addition, prenatal Poly I:C exposure led to sex-dependent alterations in QA levels and gut microbiota composition.

PPI is used to measure impairments in sensorimotor gating. Deficits in sensorimotor gating are common psychopathological characteristics and core endophenotypic markers of schizophrenia (Abel et al., 2010). The NOR test is performed to assess recognition memory (Bevins and Besheer, 2006). The EPM test and FST are used to evaluate anxiety-like behavior and depression-like behavior, respectively (Su et al., 2022; Reisinger et al., 2016). Clinical studies have shown that patients with schizophrenia of both sexes exhibit anxiety- and depression-like behaviors as well as deficits in PPI and recognition memory (Belekou et al., 2023; Kanchanatawan et al., 2018a,b; Swerdlow et al., 2018). In a ketamine mouse model of schizophrenia, chronic ketamine exposure for 12 and 14 days elicits anxiety, depression, and impairments in PPI and recognition memory in mice (Fujikawa et al., 2021; Farkhakfar et al., 2023; Xu et al., 2025). In addition, prenatal Poly I:C exposure at GD 9, 12, and 15 has been reported to trigger anxiety- and depression-like behaviors as well as recognition memory and PPI deficits in male and female offspring during adolescence and adulthood (Yildiz Taskiran et al., 2023a,b; Su et al., 2022; Reisinger et al., 2016; Osborne et al., 2019; Arad et al., 2017; Kim et al., 2018; Yang et al., 2021). In line with these findings, we observed that prenatal Poly I:C exposure at GD 9 decreased the novel object recognition index, decreased PPI, increased the immobility time in the FST, and reduced the time spent in the open arms and the number of entries to the open arms in the EPM test in adolescent and adult offspring of both sexes, suggesting impairments in recognition memory, deficits in sensorimotor gating, anxiety-like behaviors, and depression-like behaviors, respectively. By contrast, no significant differences in time spent in the center zone, number of entries to the center zone, and total distance moved in the OFT were observed between Poly I:C MIA and Veh offspring of both sexes during adolescence and adulthood. On the other hand, a previous study has indicated that administration of Poly I:C at GD 12 does not affect locomotor activity in the OFT in adult female offspring (Reisinger et al., 2016). Similarly, our results showed that Poly I:C exposure at GD 9 did not change locomotor activity in female and male offspring at adolescence and adulthood, suggesting that the impairments in recognition memory are not due to deficits in locomotor activity.

There is extensive evidence that gut microbiota dysregulation contributes to schizophrenia pathogenesis and progression (Zhu et al., 2020a,b; Zheng et al., 2019). Patients with schizophrenia exhibit abnormalities in gut microbiota composition, and these altered microbes are associated with negative and cognitive symptoms, including social function, verbal learning, visual learning, working memory, and depression (Zhu et al., 2021; Zhu et al., 2020a,b). In MK-801 mouse model of schizophrenia, mice that received an injection of MK-801 for 14 days exhibit intestinal microbiota dysfunction, anxiety and depression behaviors, and spatial recognition memory impairments, indicating that gut microbiota may be involved in these psychotic and cognitive symptoms that occur after chronic MK-801 exposure (Guo et al., 2021). Notably, mice transplanted with gut flora from schizophrenia patients show schizophrenia-like behaviors, such as increased startle responses, impaired learning and memory, and psychomotor hyperactivity (Zheng et al., 2019; Zhu et al., 2020a,b). A recent study has found that prenatal Poly I:C administration at GD 9 causes gut microbiota dysregulation in offspring of both sexes during adolescence, similar to those observed in patients with schizophrenia (Juckel et al., 2021). Moreover, prenatal Poly I:C exposure at GD 9 and 15 leads to abnormal composition of intestinal flora that may be involved in anxiety-like behaviors and impairments in recognition memory and PPI in adult male offspring (Li et al., 2021; Romero-Miguel et al., 2023). However, no attempts have been made to investigate the potential relationship between gut microbiota and prenatal Poly I:C exposure-induced schizophrenia-like abnormal behaviors in adolescent male offspring and in adolescent and adult female offspring.

In this study, our results revealed that prenatal Poly I:C exposure causes gut microbiota dysfunction, similar to that found in patients with schizophrenia. Specifically, at PND 40, families *Lachnospiraceae* and genera *Alistipes*, *Colidextribacter*, and *Lachnoclostridium* were significantly increased, while families *Muribaculaceae* and genus *Akkermansia* were significantly decreased in Poly I:C MIA offspring of both sexes. Genus *Lachnospiraceae_NK4A136_group* was significantly elevated in MIA female offspring at PND 40, but genus *Parabacteroides* was significantly reduced in MIA male offspring at PND 40. At PND 60, family *Prevotellaceae* was significantly increased only in MIA male offspring. Genus *Oscillibacter* was significantly increased only in MIA female offspring at PND 60. Genera *Lachnospiraceae_NK4A136_group* and *Ralstonia* were significantly enhanced in MIA male and female offspring at PND 60. In addition, our Pearson’s correlation analysis showed that classes *Clostridia* and genera *Colidextribacter* and *Lachnoclostridium* were significantly negatively correlated with PPI at 76 dB in offspring of both sexes at PND 40, while genus *Alistipes* was significantly negatively correlated with the novel object recognition index in offspring of both sexes at PND 40. Phylum *Firmicutes* was significantly positively associated with PPI at 85 dB, while genus *Lachnospiraceae_NK4A136_group* was significantly correlated with the novel object recognition index at in offspring of both sexes at PND 60. Furthermore, *Desulfovibrionia* at the class level and *Desulfovibrionaceae* at the family level were negatively correlated with the number of entries to open arms in male offspring at PND 40. *Helicobacter* at the genus level was positively related to immobility time in the FST at PND 40. Consistent with our findings, several recent studies have shown that the *Lachnospiraceae* and *Prevotellaceae* families, along with genera *Alistipes*, *Lachnospiraceae_NK4A136_group*, and *Lachnoclostridium,* are significantly elevated, while the *Muribaculaceae* family and genera *Akkermansia* and *Parabacteroides* are significantly reduced in schizophrenia patients and the MK-801 mouse model of schizophrenia (Liu et al., 2024; Chen et al., 2021; Yang P. et al., 2024; Yang Z. et al., 2024; Yuan et al., 2021; Nguyen et al., 2021). The *Oscillibacter* genus is significantly increased and negatively associated with working memory, visual learning, and logical memory in patients with schizophrenia (Ma et al., 2022). Meanwhile, the *Oscillibacter* genus is positively related to neuroinflammation in a rodent model of Alzheimer’s disease, and the reduction of the *Oscillibacter* genus helps to improve cognitive function and elevate learning and memory abilities (Wang et al., 2021; Wang et al., 2022). The *Alistipes* genus is a GABA-producing flora that can metabolize TRP into indole and catabolize undigested proteins into harmful metabolites such as ammonia, H_2_S, and cresol, thereby causing or aggravating depression-like behaviors (Parker et al., 2020). The *Parabacteroides* genus, a core member of the gut microbiota in both mice and humans, has physiological characteristics of carbohydrate metabolism and SCFAs secretion (Cui et al., 2022). Meanwhile, the *Parabacteroides* genus may be viewed as a potential probiotic candidate due to its protective effects on inflammation and obesity, as well as its ability to partially ameliorate anxiety- and depression-like behaviors in mice (Cui et al., 2022; Deng et al., 2021). In addition, it has been reported that increased abundance of the *Firmicutes* phylum may be implicated in learning and memory impairments and mood disorders by enhancing proinflammatory cytokine levels and modulating immune cells (Porru et al., 2024). Therefore, gut microbiota dysbiosis caused by prenatal Poly I:C exposure at GD 9 may be responsible for anxiety- and depression-like behaviors, as well as deficits in recognition memory and PPI in female and male offspring of Poly I:C MIA mothers during adolescence and adulthood.

Increasing evidence suggests that TRP and its neuroactive catabolites in systemic circulation are largely regulated by gut microbiota (Clarke et al., 2014; Zhou et al., 2023; Guo et al., 2021; Zhu et al., 2020a,b). A recent clinical study has shown that the increased abundances of gut microbiota in schizophrenia patients are negatively associated with serum TRP levels and positively associated with serum KYNA levels (Zhu et al., 2020a,b). In the MK-801 mouse model of schizophrenia, the genera *Parasutterella*, *Parabacteroides*, and *Eubacterium fissicatena* are positively correlated with brain 5-HT levels, while the genera *Lactobacillus*, *Muribaculum*, *Alistipes*, and *Lachnospiraceae_NK4A136*_group are negatively correlated with brain 5-HT levels (Guo et al., 2021). In a mouse model of depression, recolonization of the genus *Roseburia* leads to increases in 5-HT levels and decreases in 3-HK and QA levels in brain and colon tissue (Zhou et al., 2023). Compared to microbiota-rich mice, germ-free mice exhibit elevated TRP levels and reduced 5-HT and KYN levels; these abnormal alterations are reverted to normal levels after bacterial recolonization (Clarke et al., 2014). It has been reported that the genera *Alistipes* and *Lachnoclostridium* could hydrolyze TRP to indole (Parker et al., 2020; Gao et al., 2019; Sun et al., 2021). In this study, at PND 40, we found that *Firmicutes* at the phylum level was negatively associated with hippocampal 5-HT levels, but *Muribaculaceae* at the family level was positively associated with hippocampal 5-HT levels in offspring of both sexes. *Akkermansiaceae* at the family level and *Akkermansia* at the genus level were positively correlated with hippocampal KYN levels in offspring of both sexes at PND 40. *Alistipes* at the genus level was negatively associated with hippocampal KYNA levels, and *Lachnoclostridium* at the genus level was positively correlated with hippocampal QA levels in offspring of both sexes at PND 40. *Firmicutes* at the phylum level was positively associated with hippocampal 5-HT levels, and *Lachnospiraceae_NK4A136_group* at the genus level was negatively associated with fecal TRP levels in offspring of both sexes at PND 60. In addition, *Oscillospiraceae* at the family level was negatively correlated with serum TRP levels in male offspring at PND 40. *Eggerthellaceae* at the family level was positively associated with serum QA levels, while *Parabacteroides* at the genus level was negatively associated with serum QA levels in male offspring at PND 40. *Helicobacter* at the genus level was positively related to serum QA levels in female offspring at PND 40. *Verrucomicrobiae* at the class level, *Verrucomicrobiales* at the order level, and *Akkermansiaceae* at the family level were positively associated with serum TRP and KYN levels in female offspring at PND 60. *Oscillospiraceae* at the family level showed a negative correlation with fecal TRP levels in female offspring at PND 60. Moreover, *Oscillibacter* at the genus level was negatively related to fecal KYN levels and positively related to fecal QA levels in female offspring at PND 60. Taken together, these findings reveal that gut microbiota plays an important role in regulating the TRP metabolism pathway.

It has been reported that the imbalance of the TRP metabolism pathway is associated with psychotic and cognitive symptoms in schizophrenia (Kanchanatawan et al., 2018a,b; Cathomas et al., 2021; Chen et al., 2024). As an excitotoxin, increased QA levels overactivate the NMDA receptor and increase the release of glutamate, leading to neurodegeneration, neuronal damage, and thus impairing brain function (Cao et al., 2021). Clinical studies have found that patients with schizophrenia show increased QA levels and reduced TRP, KYN, and KYNA levels in serum and plasma, and that elevated QA levels are correlated with cognitive deficits, including attention, executive function, language function, and visual learning (Cathomas et al., 2021; Chen et al., 2024). It has been reported that increased QA levels in plasma are involved in the pathogenesis of negative symptoms in schizophrenia (Kanchanatawan et al., 2018a,b). Brain injection of QA significantly elicits impairments in PPI and recognition memory in rats (Velloso et al., 2009; Shoemaker et al., 2003). Moreover, acute lipopolysaccharide (LPS) injection increases QA levels in the brain and plasma in mice, which may be correlated with LPS-induced depression- and anxiety-like behaviors (Verdonk et al., 2019). On the other hand, the lack of 5-HT in the central nervous system (CNS) affects postnatal CNS development and proper wiring of the brain, leading to brain dysfunction (Migliarini et al., 2013). A significant decrease in 5-HT levels in full blood is found in schizophrenia patients, and this decrease in 5-HT levels is negatively correlated with depressive symptoms in patients (Peitl et al., 2016). In the MK-801 rat model of schizophrenia, reduced frontal cortex levels of 5-HT are significantly associated with impairments in recognition memory in rats (Hereta et al., 2020). A previous animal study has indicated that reduced levels of median raphe nucleus 5-HT by a 5-HT neurotoxin will lead to deficits in PPI in rats (Kusljic et al., 2006). In a mouse model of constipation, decreased levels of serum 5-HT may be implicated in constipation-induced anxiety- and depression-like behaviors in mice (Zou et al., 2024). In addition, Poly I:C-induced MIA increases mRNA levels of KMO and the QA/KYNA ratio and decreases 5-HT levels in the frontal cortex in adult offspring of both sexes (MacDowell et al., 2021). Poly I:C MIA elevates QA levels and reduces KYN and KYNA levels in serum in adolescent male offspring (Zavitsanou et al., 2014). Increased brain serotonergic activity by selective 5-HT reuptake inhibitors improves impairments in PPI and recognition memory in adult male offspring of dams exposed to Poly I:C during gestation (Taskiran et al., 2024). Prenatal Poly I:C exposure increases 3-HK levels in the placenta and fetal brain, and this increase in 3-HK levels is correlated with impairment in recognition memory in adult male offspring (Hasegawa et al., 2024). In the present study, we found that prenatal Poly I:C exposure at GD 9 reduced serum and hippocampal levels of TRP, 5-HT, KYN, and KYNA, increased serum QA in female offspring at adolescence and adulthood, and increased hippocampal levels of QA in male and female offspring at adolescence and adulthood. In addition, we found that prenatal Poly I:C exposure at GD 9 increased hippocampal expression of IDO1 and KMO but decreased hippocampal expression of TPH2 and KATII in female and male offspring at adolescence and adulthood. Moreover, prenatal Poly I:C exposure at GD 9 decreased fecal levels of TRP, 5-HT, and KYN in female and male offspring at adolescence and adulthood and increased fecal QA levels in male and female offspring at adolescence but only in females at adulthood. Our results suggest that prenatal Poly I:C exposure at GD 9 facilitates the shift of TRP metabolism from the formation of KYNA and 5-HT toward the production of QA in offspring of both sexes at adolescence and adulthood. Together, these findings suggest that the upregulation of the TRP-KYN-QA pathway induced by gut dysbiosis may be responsible for anxiety- and depression-like behaviors as well as deficits in recognition memory and PPI during adolescence and adulthood in female and male offspring of Poly I:C MIA mothers.

It has been reported that Poly I:C binds to TLR3 to activate proinflammatory signaling, increasing proinflammatory cytokine levels that can cross the placental barrier and blood-brain barrier, thus leading to peripheral inflammation and neuroinflammation in offspring (Su et al., 2022). Proinflammatory cytokines are known inducers of IDO1 and KMO expression (Kindler et al., 2020), suggesting that an increased inflammatory response may activate the QA pathway. In addition, the *Muribaculaceae* family, a member of the *Bacteroidetes* order, has been reported to metabolize both endogenous and exogenous polysaccharides into SCFAs, thereby exerting anti-inflammatory effects (Zhu et al., 2024). Intestinal colonization of the *Akkermansia* genus has a close relationship with host health. The *Akkermansia* genus can effectively improve gut barrier function by increasing the thickness of the intestinal mucus layer and can a produce beneficial immune response by modulating immune cells (Cani et al., 2022). The *Colidextribacter* genus is involved in the modulation of inflammation markers, and the decrease in the abundance of the *Colidextribacter* genus may be beneficial in alleviating the severity of neuroinflammation (Yang P. et al., 2024; Yang Z. et al., 2024). It has been reported that the increased abundance of the *Ralstonia* genus leads to the overproduction of LPS and activation of the TLR4-NF-κB signaling pathway, which in turn triggers an inflammatory response (Zhang et al., 2024). Furthermore, the *Lachnospiraceae* family, a member of the *Firmicutes* phylum, could recruit macrophages and promote the translocation of LPS from the gastrointestinal tract into the systemic circulatory system, thereby elevating proinflammatory cytokine levels in the peripheral and central nervous system, and causing an immune inflammatory response (Sorbara et al., 2020; Yang et al., 2023). The *Lachnospira ceae_NK4A136_group* genus induces gastrointestinal inflammation by activating the TLR4-NF-κB pathway and is positively correlated with the pathological characteristics of colitis in mice (He et al., 2023). A recent animal study has found that the *Prevotellaceae* family, as Gram-positive bacteria, can induce a systemic inflammatory response by upregulating the sphingosine-1-phosphate receptor 2-NF-κB signaling pathway in mice (Chen et al., 2025). Meanwhile, the study also showed that the increased abundance of the *Prevotellaceae* family may be promote the colonization of pathogenic bacteria (Chen et al., 2025). In addition, the *Clostridia* class is an important producer of biotoxins and can secrete various toxins that induce apoptosis and interfere with nerve signal transmission (Philippe et al., 2006). Meanwhile, the *Clostridia* class could also lead to systemic infections (Morvan et al., 2021). These findings suggest that gut microbiota play a key role in the regulation of inflammatory responses. Thus, the upregulation of the QA pathway in our study may be partly caused by the enhanced inflammatory response mediated by gut microbiota dysbiosis that occurs after prenatal Poly I:C exposure.

Previous studies have found that there are significant differences in estrogen levels between female and male mice, with estrogen levels being higher in female mice than in male mice (Gegenhuber et al., 2022; Du et al., 2024). Estrogen has been reported to enhance the expression of IDO1 and inhibit KATII activity (Jayawickrama et al., 2017; Li et al., 2020), which may activate the TRP-KYN-3-HK pathway, leading to increases in QA levels. Young women who take oral contraceptives (generally containing both estrogen and progesterone analogues) exhibit increased excretion of KYN pathway metabolites, including 3-HK, 3-hydroxyanthranilic acid, and QA (de Bie et al., 2016). A previous study indicates sex-dependent differences in the neuroinflammatory responses of offspring induced by prenatal Poly I:C exposure (Posillico et al., 2021). Type I interferons are important to the anti-viral response and respond to viral stimulants such as Poly I:C (Posillico et al., 2021). It has been reported that type I interferons can facilitate IDO1 expression, thereby activating the TRP-KYN metabolism pathway (Zhu et al., 2020a,b). More importantly, the expression of type I interferons is significantly different in male and female offspring of pregnant dams injected with Poly I:C, suggesting a specific sex-related difference in the anti-viral response (Posillico et al., 2021). In this study, we revealed that prenatal Poly I:C exposure increased hippocampal QA levels in offspring of both sexes at PND 40 and 60, with hippocampal QA levels being higher in female offspring than in male offspring. Meanwhile, serum QA levels were elevated only in female MIA offspring at PND 40 and 60. Furthermore, fecal QA levels were significantly elevated only in MIA female offspring at PND 60. Our results revealed that the effect of prenatal Poly I:C exposure on the QA metabolism pathway is more pronounced in female offspring, suggesting sex-related differences in the TRP-KYN-QA pathway. In addition, estrogen has been shown to have a profound impact on gut microbiota composition (Dominianni et al., 2015; Juckel et al., 2021). Previous studies have found that microbiome composition differs significantly between males and females in humans as well as rodents (Dominianni et al., 2015; Juckel et al., 2021). In this study, we found that prenatal Poly I:C exposure increased the abundance of classes *Desulfovibrionia*, orders *Clostridia_UCG-014*, and families *Desulfovibrionaceae*, *Oscillospiraceae*, and *Eggerthellaceae*, decreased the abundance of genera *Parabacteroides* in male MIA offspring, and increased the abundance of genera *Lachnospiraceae_NK4A136_group* and *Helicobacter* in female MIA offspring at PND 40. Furthermore, prenatal Poly I:C exposure increased the abundance of families *Prevotellaceae* and *Bacteroidaceae* and genera *Bacteroides*, decreased the abundance of genera *Desulfovibrio* in male MIA offspring, elevated the abundance of *Oscillospiraceae* and genera *Oscillibacter*, and reduced the abundance of classes *Verrucomicrobiae*, orders *Verrucomicrobiales*, and families *Akkermansiaceae* in female MIA offspring at PND 60. Our results showed that prenatal Poly I:C exposure has different effects on the composition of gut microbiota in female and male offspring, suggesting sex-dependent differences in gut microbiota composition. Together, our study provides a foundation for further research to clarify whether sex-related TRP-KYN-QA metabolism pathways and gut microbiota changes caused by MIA could contribute to behavioral domains not detected here, or might confer susceptibility to – or protect against – double-hit effects.

Increasing evidence suggests that gut microbiota disorder is implicated in psychotic symptoms and cognitive impairments in patients with schizophrenia (Zhu et al., 2021; Zhu et al., 2020a,b). It has been reported that gut microbiota dysregulation impairs neurodevelopment, neurogenesis, and synaptic structure and function, affects neurotransmitter metabolism, and changes the levels of microbial metabolites, thereby causing psychotic symptoms and cognitive deficits in schizophrenia (Zhu et al., 2020a,b; Guo et al., 2021; Cussotto et al., 2018). Further studies are necessary to explore whether gut microbiota dysbiosis leads to Poly I:C MIA-induced anxiety- and depression-like behaviors, as well as PPI and recognition memory deficits by affecting nervous system development, synaptic plasticity, neurotransmitter metabolism, and microbial metabolite levels. In addition, given the intricate composition and structure of gut microbiota, the connection between aberrant abundance of specific bacterial genera and psychotic and cognitive symptoms remains unclear. Further studies, such as fecal microbiota transplant (FMT) experiments or probiotics/prebiotics interventions, are warranted to elucidate the potential mechanisms by which certain bacterial alterations contribute to Poly I:C MIA-induced psychotic symptoms and cognitive impairments. Furthermore, estrogen has been reported to affect the TRP-KYN metabolism pathway and the composition and structure of gut microbiota (Jayawickrama et al., 2017; Dominianni et al., 2015). Further studies are necessary to investigate whether estrogen is involved in Poly I:C MIA-caused psychotic and cognitive symptoms by mediating the TRP-KYN metabolism pathway and gut microbiota.

## 5 Conclusion

In summary, our results provide the first molecular and behavioral evidence that gut dysbiosis induced by prenatal Poly I:C exposure is likely responsible for prenatal Poly I:C exposure-induced anxiety- and depression-like behaviors and impairments in PPI and recognition memory by disrupting the TRP metabolism pathway in adolescent and adult offspring of both sexes. Our study suggests possible strategies to alleviate prenatal Poly I:C exposure-associated schizophrenia-like behaviors. Our findings provide additional evidence that gut microbiota dysbiosis is the mechanism underlying Poly I:C MIA-associated schizophrenia-like behaviors and behavioral deficits in schizophrenia. Considering the sex-related differences in the gut microbiota and TRP-KYN-QA pathway, both sexes should be included in the studies that investigate the mechanisms underlying Poly I:C MIA-associated schizophrenia-like behaviors.

## Data Availability

The raw data supporting the conclusions of this article will be made available by the authors, without undue reservation.
