# Supplementary material for: Poly I:C-induced maternal immune activation causes schizophrenia-like behaviors in the offspring of both sexes by regulating gut microbiota and tryptophan metabolism pathway
Source: Front Microbiol. 2025 Sep 30;16:1667164. doi: 10.3389/fmicb.2025.1667164 (PMC12520628; doi:10.3389/fmicb.2025.1667164)
Supplement: Supplementary file 1 [file Supplementary_file_1.docx]

***Supplementary materials***

**1 Antibodies used in Western blot analysis**

The following primary antibodies were used: anti-TPH2 (Abcom, Cambridge, UK, ab184505; 1:1000), anti-IDO1 (FabGennix, Boston, USA, IDO1-101AP; 1:2500), anti-KMO (Thermo Scientific, Rockford, USA, PA5-100019; 1:500), anti-KATⅡ (Santa Cruz, Boston, USA, SC-365847; 1:100), and anti-β-actin (CST, Boston, USA, 4970; 1:1000). The following HRP-conjugated secondary antibody was used: anti-rabbit IgG (H+L) (CST, Boston, USA, 7074; 1:3000); anti-mouse IgG（H+L）(CST, Boston, USA, 7076; 1:3000).

**2 Primer used in Real-time quantitative PCR (RT-qPCR)**

The specific RT-qPCR primer sequences were as follows:

TPH2: forward 5′-CACCGAGTCCTCATGTACGG-3′,

reverse 5′-GCAAGCATGAGTCGGGTAGA-3′;

IDO1: forward 5′-GGATCCTTGAAGACCACCACAT-3′,

reverse 5′-AAGGACCCAGGGGCTGTAT-3′;

KMO: forward 5′-ATGGCATCGTCTGATACTCAGG-3′,

reverse 5′-CCCTAGCTTCGTACACATCAACT-3′;

KATⅡ: forward 5′-GTTCTCCACACACAAGTCTC-3′,

reverse 5′-GGATCCATCCTGTCAGTCA-3′;

GAPDH: forward 5′-ACCACAGTCCATGCCATCAC-3′,

reverse 5′-ACCTTGCCCACAGCCTTG-3′.


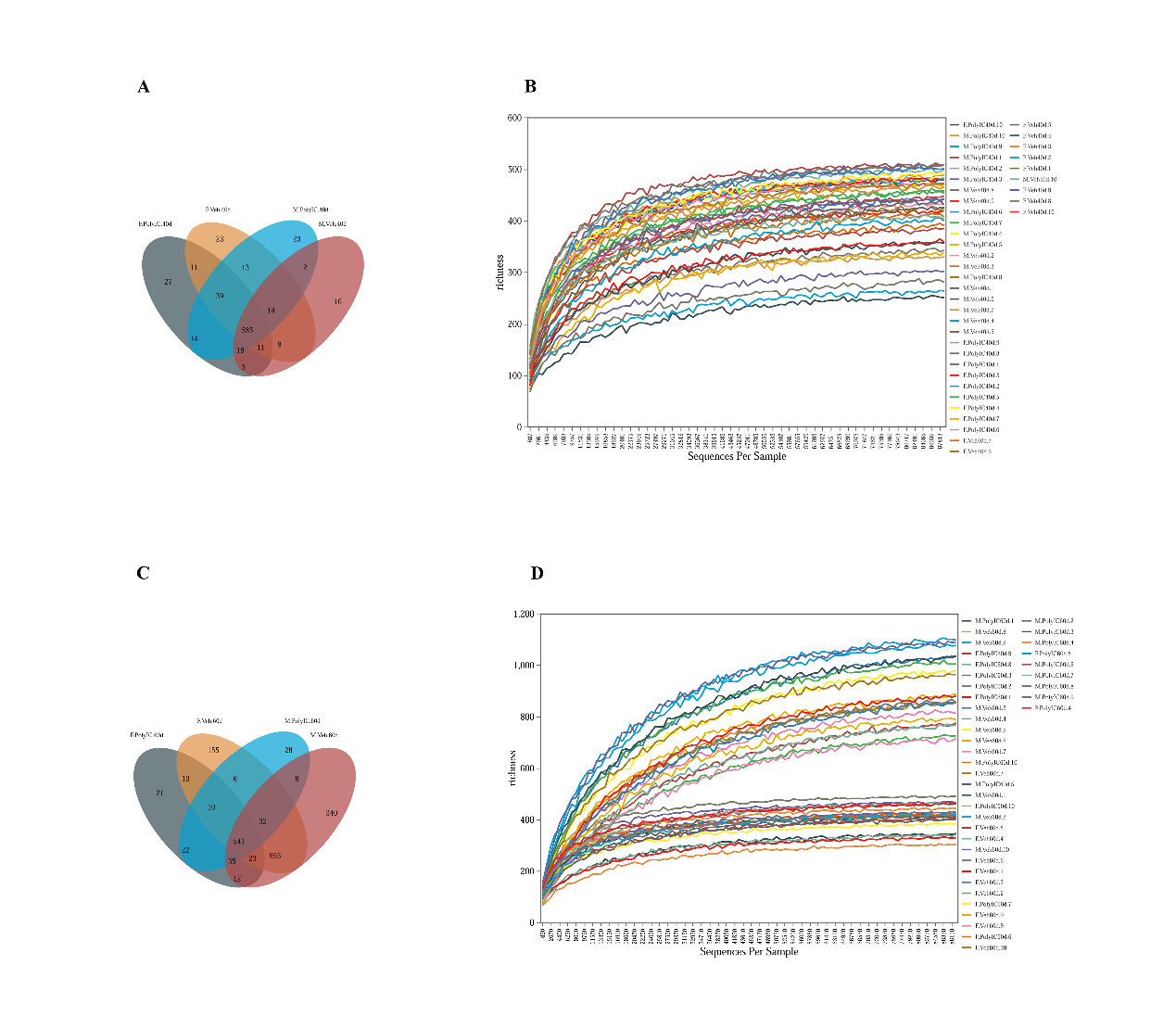


**Supplementary Figure 1.** Venn diagram and rarefaction curve analyses of microbial community overlap and sequencing depth adequacy. (**A**) A Venn diagram showed that 585 of the 813 OTUs were commonly identified in the four groups, while 16, 23, 29, and 27 were unique in Veh male, Veh female, MIA male, and MIA female offspring at PND 40, respectively (n = 10). (**C**) Venn diagram showed that 541 of the 2, 236 OTUs were commonly detected among groups, while 340, 155, 28, and 21 were unique in Veh male, Veh female, MIA male, and MIA female in offspring at PND 60, respectively (n = 10). (**B**, **D**) The majority rarefaction curves approached the saturation plateau, suggesting that the sequencing depth of gut microbiota was adequate (n = 10). Veh, vehicle; MIA, maternal immune activation; OTUs, operational taxonomic units.
